# PGE_2_/EP3/SRC signaling induces EGFR nuclear translocation and growth through EGFR ligands release in lung adenocarcinoma cells

**DOI:** 10.18632/oncotarget.16116

**Published:** 2017-03-10

**Authors:** Lorenzo Bazzani, Sandra Donnini, Federica Finetti, Gerhard Christofori, Marina Ziche

**Affiliations:** ^1^ Department of Life Sciences, University of Siena, 53100, Siena, Italy; ^2^ Department of Biomedizin, University of Basel, 4058, Basel, Switzerland

**Keywords:** nuclear EGFR, PGE_2_, EP3, EGFR ligands, lung cancer

## Abstract

Prostaglandin E_2_ (PGE_2_) interacts with tyrosine kinases receptor signaling in both tumor and stromal cells supporting tumor progression. Here we demonstrate that in non-small cell lung carcinoma (NSCLC) cells, A549 and GLC82, PGE_2_ promotes nuclear translocation of epidermal growth factor receptor (nEGFR), affects gene expression and induces cell growth. Indeed, cyclin D1, COX-2, iNOS and c-Myc mRNA levels are upregulated following PGE_2_ treatment. The nuclear localization sequence (NLS) of EGFR as well as its tyrosine kinase activity are required for the effect of PGE_2_ on nEGFR and downstream signaling activities. PGE_2_ binds its *bona fide* receptor EP3 which by activating SRC family kinases, induces ADAMs activation which, in turn, releases EGFR-ligands from the cell membrane and promotes nEGFR. Amphiregulin (AREG) and Epiregulin (EREG) appear to be involved in nEGFR promoted by the PGE_2_/EP3-SRC axis. Pharmacological inhibition or silencing of the PGE_2_/EP3/SRC-ADAMs signaling axis or EGFR ligands i.e. AREG and EREG expression abolishes nEGFR induced by PGE_2_. In conclusion, PGE_2_ induces NSCLC cell proliferation by EP3 receptor, SRC-ADAMs activation, EGFR ligands shedding and finally, phosphorylation and nEGFR. Since nuclear EGFR is a hallmark of cancer aggressiveness, our findings reveal a novel mechanism for the contribution of PGE_2_ to tumor progression.

## INTRODUCTION

Aberrant growth signals in malignant tumors, including non small cell lung cancer (NSCLC) are frequently due to the deregulation of signaling cascades of growth factors and their receptors, such as epidermal growth factor receptor (EGFR) and its ligands [1]. The tumor microenvironment actively contributes to these events by providing cellular and molecular effectors which enhance the dysregulation of cancer cell signaling [2].

Prostaglandin E_2_ (PGE_2_), an inflammatory mediator, initiates multiple cellular responses, including tumor cell growth and progression. Increased PGE_2_ synthesis was observed in different malignancies such as colon, breast, lung, head and neck, prostate and bladder cancer [3, 4]. Notably, cycloxigenase-2 (COX-2) and microsomal prostaglandin E synthase-1 (mPGES-1), the two inducible key enzymes in PGE_2_ biosynthesis, were found overexpressed in NSCLC, correlating with the reduced survival in patients with stage I disease [5–7]. In addition to the large bulk of literature on prostanoids and colon cancer, several studies have shown that NSAID and aspirin reduced the risk to develop lung cancer [8, 9].

We and others have previously reported the importance of PGE_2_ in several processes of tumor cell adaptation to the microenvironment, such as cell survival, growth, migration, invasion, and angiogenesis [10–15]. In addition, PGE_2_ can transactivate EGFR-mediated signaling networks that confer an aggressive phenotype to tumor cells [16–18]. More recently, PGE_2_ has also been identified as a tumor-induced immunosuppressive factor, able to mediate the reprogramming of the tumor microenvironment [19], or as a direct modulator of macrophage activity by transactivation of CSF-1R [20]. All together, these data highlight the complex effects exerted by PGE_2_ on stromal/immune and cancer cells in creating a pro-tumorigenic microenvironment and in supporting tumor progression.

Using different experimental models, several reports have demonstrated EGFR activation by PGE_2_ and its receptors (EP receptors) coupled to different downstream effectors, including PKA, PKC, SRC and PI3K [17, 18, 21, 22]. The best-characterized mechanisms by which PGE_2_/EP signaling transactivates EGFR involve the autocrine and/or paracrine release of soluble EGF-like ligands [23]. Ligand shedding-independent transactivation of EGFR by direct intracellular phosphorylation has also been proposed [17, 18, 24, 25]. In this context, EGFR-supported transactivation is strongly dependent on intracellular signaling pathways, such as Ca^2+^, PKC and the non-receptor tyrosine kinase c-SRC [26].

Ligand binding to EGFR induces a variety of signaling cascades from the plasma membrane to different subcellular compartments [27]. Notably, ligand-activated EGFR can be targeted to the nucleus, where it acts as transcription factor and chromatin regulator and affects gene expression, DNA replication, and DNA damage repair promoting tumor progression, aggressiveness and resistance to therapies [28, 29]. In lung adenocarcinoma, nuclear EGFR expression has been associated with poor clinical outcome and chemo-resistance [30].

Despite the experimental evidence on the functional interaction between PGE_2_ and EGFR, the role of PGE_2_ in EGFR nuclear translocation is not known. Since we have previously demonstrated that PGE_2_ induces angiogenesis by promoting fibroblast growth factor receptor-1 (FGFR1) nuclear translocation [31] and that PGE_2_ transactivates EGFR leading to tumor progression [15, 17], we have tested whether PGE_2_ coupling with EP receptors induces EGFR nuclear shuttling in NSCLC cells. Here we report the molecular mechanisms by which PGE_2_ regulates EGFR nuclear translocation and the contribution of this signaling cascade to sustain tumor growth.

## RESULTS

### PGE_2_ promotes EGFR nuclear translocation and cell growth in human NSCLC cells

**Figure 1:**
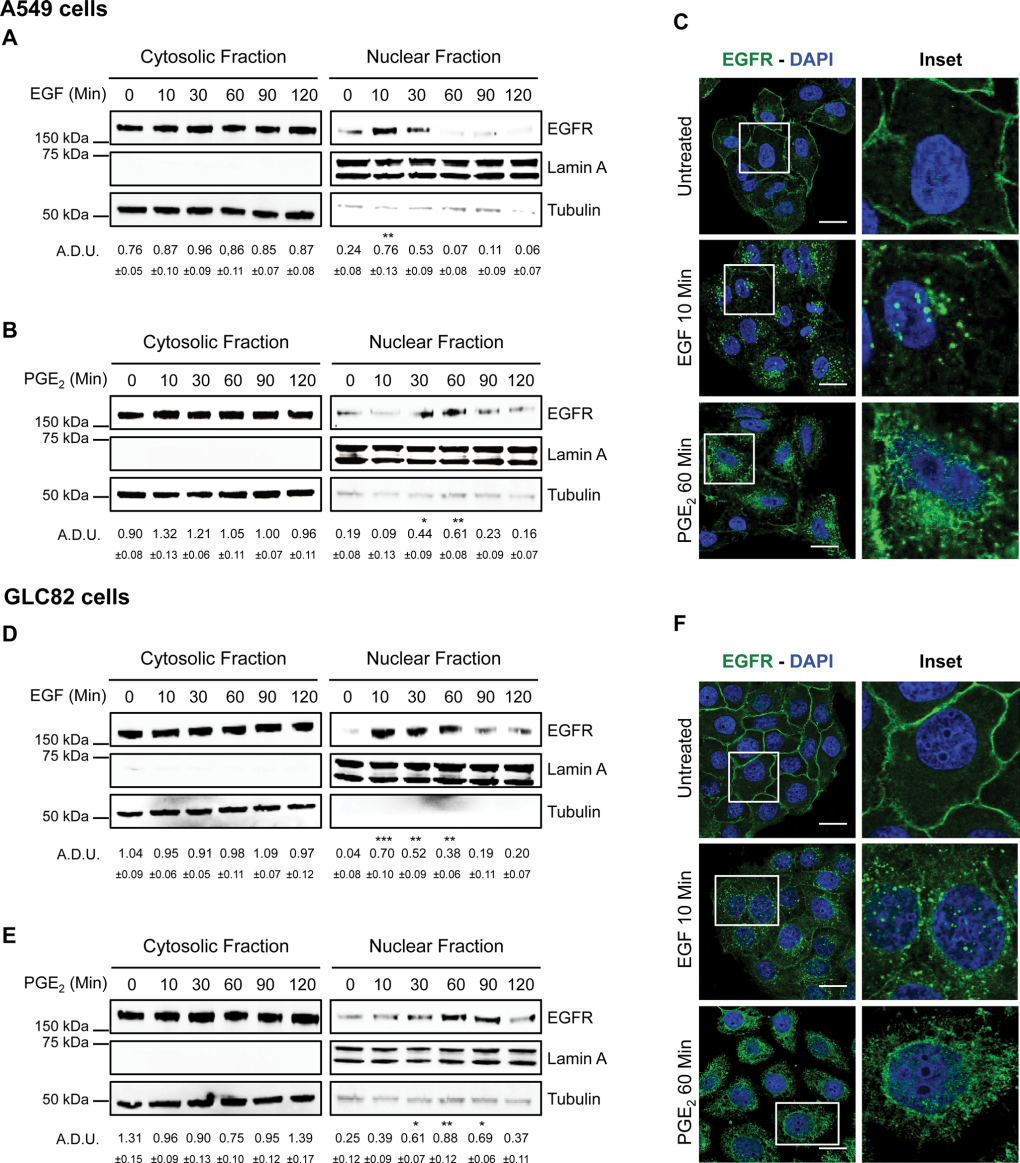
PGE_2_ induces EGFR nuclear translocation Immunoblotting analysis of EGFR expression in cytosolic and nuclear fraction in overnight starved A549 (**A**, **B**, **C**) and GLC82 (**D**, **E**, **F**). Cells were exposed for 10–120 min to 25 ng/ml EGF (A, D) or 1 μM PGE_2_ (B, E). Tubulin and Lamin A were used as loading control for cytosolic and nuclear fraction respectively. Immunoblotting quantification was expressed in A.D.U. (arbitrary density unit) and as mean ± SEM. *p < 0.05, **p < 0.01, ***p < 0.001 vs Ctrl. EGFR in the cytoplasmic and nuclear fractions was normalized to Tubulin or Lamin A respectively. Confocal analysis of EGFR localization in A549 (C) and GLC82 (F) exposed to 25 ng/ml EGF (10 min, middle panel) or 1 μM PGE_2_ (60 min, bottom panel). EGFR was stained in green and DAPI (blue) was used to counterstain the nuclei. Confocal images were captured in the middle section of the nuclei using 63× objective. Scale bars, 20 μm. Boxed areas are shown in detail in the inset.

Using A549 and GLC82 NSCLC cells, we investigated whether PGE_2_ promoted EGFR nuclear internalization. EGF was used as a positive control. Cells were treated with EGF 25 ng/ml (10–120 min), and EGFR nuclear translocation was determined by cell fractionation and immunoblotting. Upon EGF treatment, EGFR translocated to the nucleus with a peak at 10 min and declined to baseline at 60 min (Figure 1A for A549 cells and 1D for GLC82 cells). To assess whether PGE_2_ was able to induce EGFR nuclear translocation, we treated tumor cells for the same time points with PGE_2_ 1 μM. PGE_2_ induced EGFR nuclear accumulation, which was detectable starting at 30 min, reaching a plateau at 60 min and declining toward the baseline at 120 min after treatment (Figure 1B and 1E). Immunofluorescence staining followed by confocal microscopy analysis showed that in control conditions, EGFR was confined to the cell membrane (Figure 1C and 1F and *upper panels*). After 10 min of EGF 25 ng/ml treatment, EGFR was mobilized from the cell membrane and localized within the nucleus, an event reproduced by 60 min exposure to PGE_2_ 1 μM (Figure 1C and 1F *central* and *bottom* panels, respectively). 3D reconstruction of confocal laser scanning microscopy stacks confirmed the nuclear translocation of EGFR upon EGF or PGE_2_ treatment (Supplementary Figure 1A and 1B).

**Figure 2:**
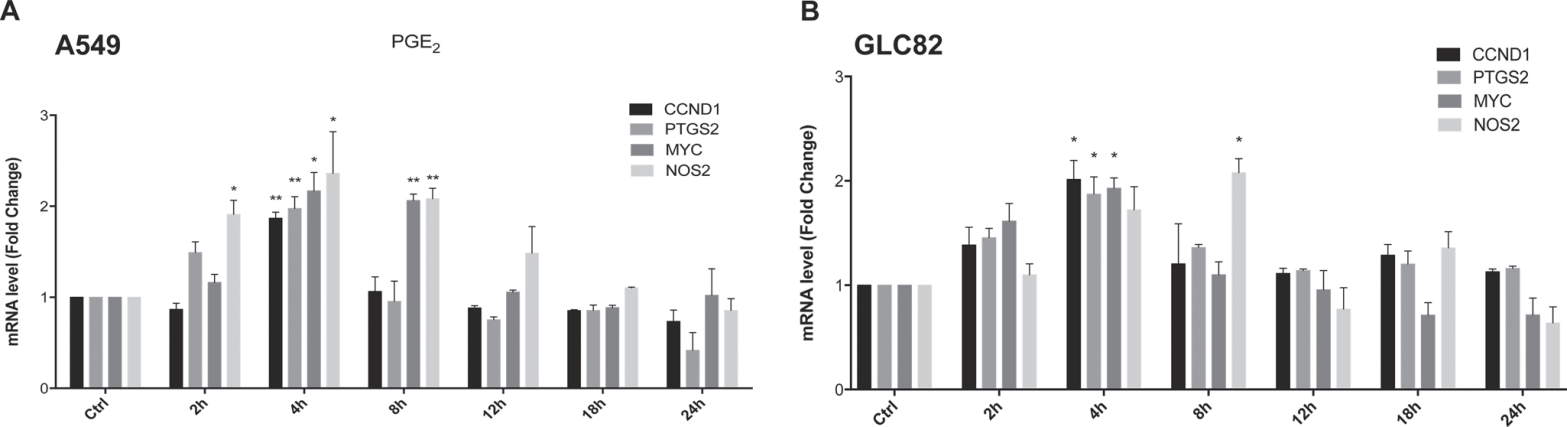
PGE_2_ regulates nuclear EGFR target genes A549 (**A**) and GLC82 (**B**) cells were starved overnight and then treated with 1 μM PGE_2_ for 2, 4, 8, 12, 18, 24 h. RNA was isolated and analyzed by qRT-PCR for a panel of nuclear EGFR target genes. The data are presented as mean of fold change ± SEM of three independent experiments, relative to non-treated cells (Control), which were assigned to 1. *p < 0.05, **p < 0.01 vs Ctrl.

Next, we investigated whether the PGE_2_-mediated EGFR nuclear internalization was associated with increased cell growth. In A549 cells exposed for a time frame of 2–24 h to the treatments, EGF promoted the expression of a panel of well-known nuclear EGFR-target genes involved in cell proliferation, cell cycle progression and inflammation, such as cyclin D1 (*CCND1*), c-Myc (*MYC*) cyclooxygenase-2 (*PTGS2*), and inducible nitric oxide synthase (*NOS2*) (Supplementary Figure 2A), maximal activation occurred at 2 h. PGE_2_ mimicked EGF activity on nuclear EGFR-target gene expression with a maximal effect at 4 h in both A549 and GLC82 cells (Figure 2A and 2B). Other nuclear EGFR-target genes, such as Aurora A (*AURKA*), Breast cancer resistant protein (*BCRP*), B-Myb (*MYBL2*) and Thymidylate synthase (*TYMS*), were not regulated by EGF or PGE_2_ (Supplementary Figure 2B, 2C and 2D).

**Figure 3:**
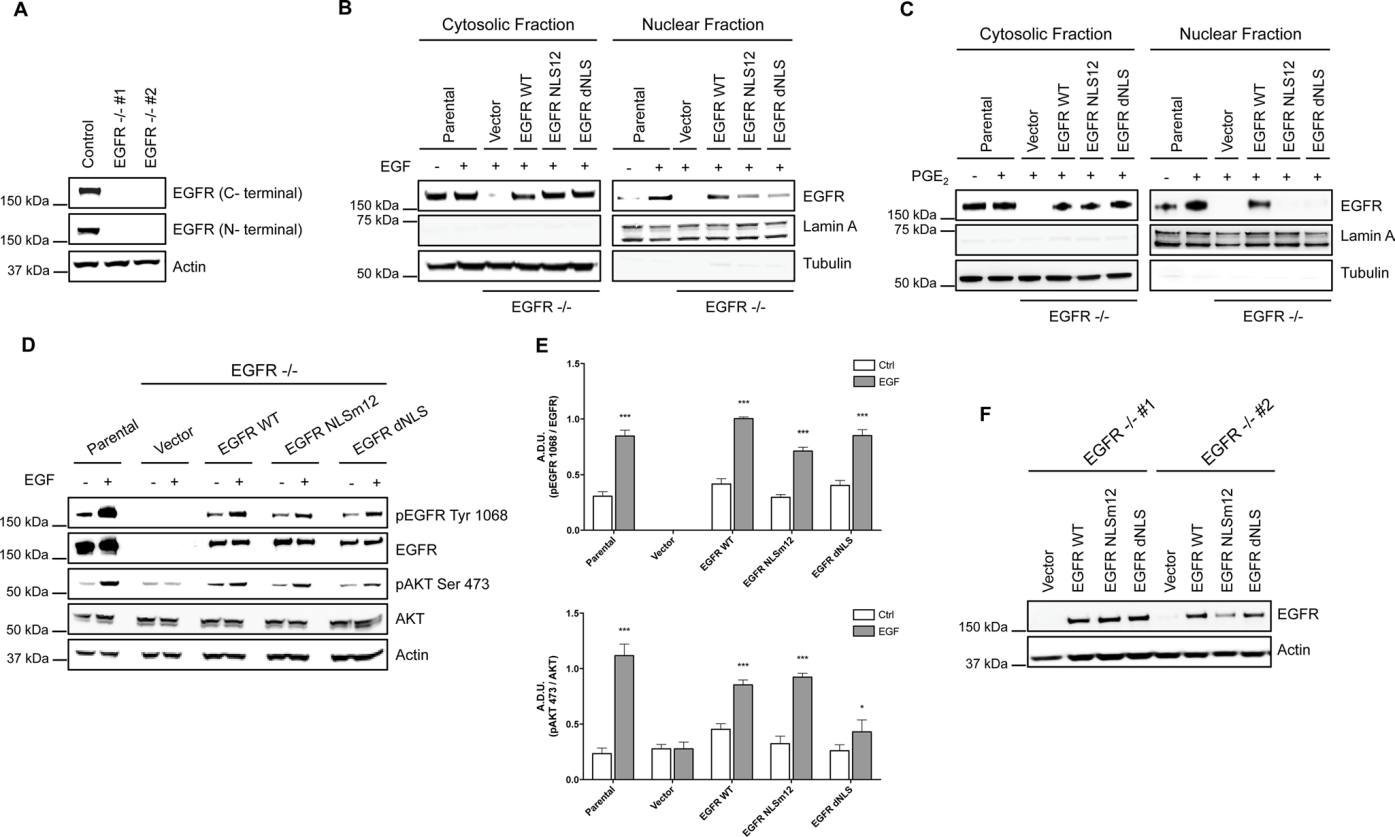
NSCLC cell models to study PGE_2_-induced EGFR nuclear translocation (**A**) Immunoblotting analysis of EGFR expression in A549 wild type cells and two clones knockout for EGFR, generated by CRISPR/Cas9 (EGFR −/− #1, #2). Actin was used as loading control. (**B**, **C**) EGFR knockout cells were transiently transfected with Vector or EGFR-WT or EGFR mutated in NLS (NLSm12 or dNLS) plasmids for 48 h. Then EGFR nuclear translocation in response to 25 ng/ml EGF for 10 min (B) or 1 μM PGE_2_ for 60 min (C) was analyzed by immunoblotting upon cell fractionation. Parental cells were included as a control. Tubulin and Lamin A were used as loading control for cytosolic and nuclear fraction respectively. (**D**) Immunoblotting analysis of EGFR phosphorylation on tyrosine 1068 and AKT on serine 473, upon EGF treatment in parental and EGFR knockout cells expressing Vector, EGFR-WT and NLS mutant plasmids. (**E**) Immunoblotting quantification of pEGFR Tyr 1068, normalized to EGFR and pAKT Ser 473, normalized to AKT, were expressed in A.D.U. (arbitrary density unit) and as mean ± SEM. *p < 0.05, ***p < 0.001 vs Ctrl. (**F**). Expression of EGFR in EGFR −/− #1, #2 transfected with Vector, EGFR-WT and NLS mutant plasmids for 72 h.

**Figure 4:**
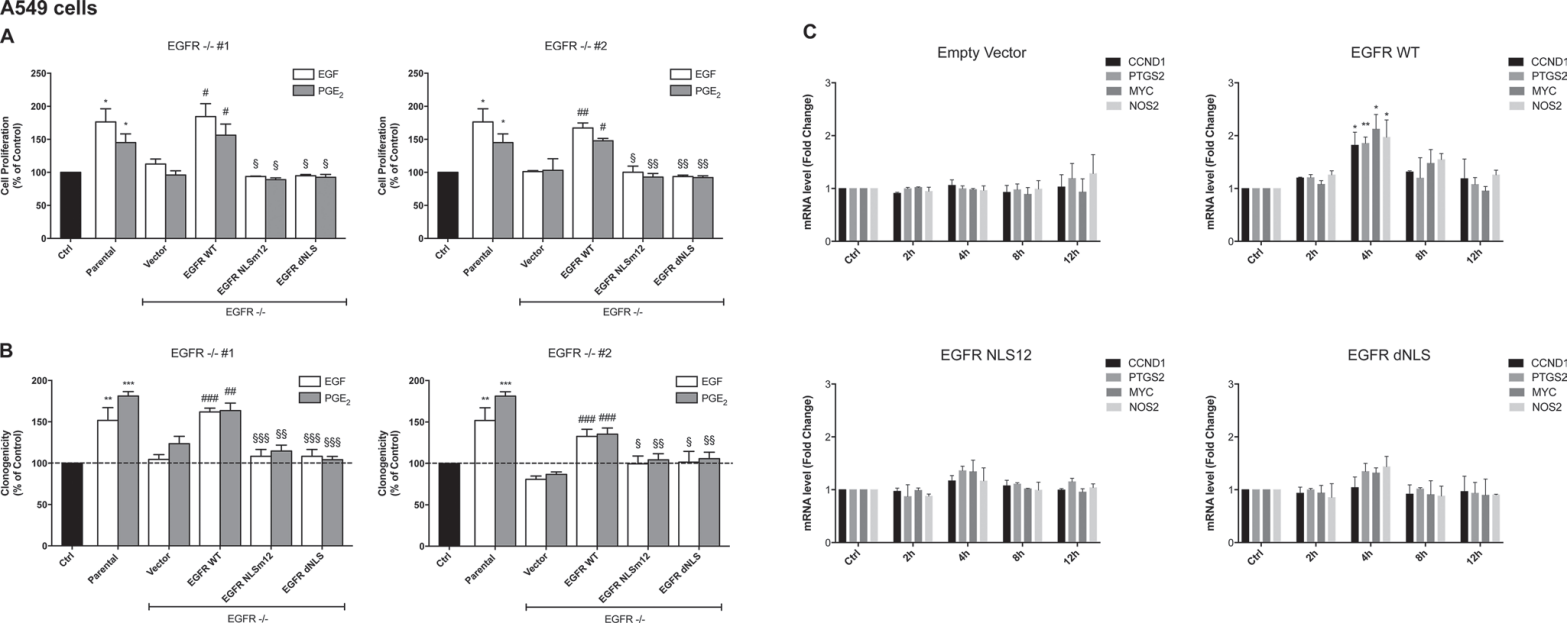
PGE_2_ promotes cell proliferation, clonogenicity and gene regulation via nuclear EGFR (**A**, **B**, **C**) Parental A549 cells or EGFR −/− #1, #2 cells transfected with Vector or EGFR-WT or NLS mutant plasmids, were seeded and incubated for 24 h. Next, cells were harvested and seeded for MTT, clonogenic assay and RNA isolation. (A) Cell growth was assessed by MTT assay after 48 h treatment with 25 ng/ml EGF or 1 μM PGE_2_. Data are presented as mean ± SEM of triplicate cultures, expressed as % of control. *p < 0.05 vs Ctrl; ^#^*p* < 0.05, ^##^*p* < 0.01 vs Vector; ^§^*p* < 0.05, ^§§^*p* < 0.01 vs EGFR WT. (B) Clonal outgrowth was assessed by counting number of clones (>50 cells) 12 days after treatment with 25ng/ml EGF or 1μM PGE_2_. Data are presented as mean ± SEM of triplicates, expressed as % of control. **p < 0.01, ***p < 0.001 vs Ctrl; ^##^*p* < 0.01, ^###^*p* < 0.001 vs Vector; ^§^*p* < 0.05, ^§§^*p* < 0.01, ^§§§^*p* < 0.001 vs EGFR WT. (C) RNA was isolated after 2, 4, 8, 12 h treatment with 1μM PGE_2_ and analyzed by qRT-PCR for regulated nuclear EGFR target genes. The data are presented as fold change ± SEM of three independent experiments, relative to non-treated cells (Control), which were assigned to 1. *p < 0.05, **p < 0.01 vs Ctrl.

To demonstrate that the tumor gene reprogramming promoted by PGE_2_ was mediated by nuclear EGFR, the expression of EGFR was genetically ablated by CRISPR/Cas9 in A549 (Figure 3A) and GLC82 cells (Supplementary Figure 3A), and then two clones, knockout for EGFR (EGFR −/− #1, #2), were transfected with EGFR plasmids bearing a wild type (WT) or a mutated nuclear localization sequence, NLSm12 and dNLS, respectively [32]. In NLSm12 and dNLS cells, EGFR nuclear translocation by either EGF or PGE_2_ was significantly reduced compared to cells expressing WT EGFR or to parental cells (Figure 3B and 3C). EGFR-NLS clones maintained the EGF-induced EGFR canonical signaling, such as receptor phosphorylation on Tyr 1068 and AKT activation, as did the EGFR WT clones (Figure 3D and 3E). Further, A549 and GLC82 cells transfected with constructs encoding for WT and mutant EGFR exhibited a comparable level of EGFR expression (Figure 3F and Supplementary Figure 3B), yet only cells expressing WT EGFR showed significant cell proliferation when exposed to EGF or PGE_2_, while cells expressing EGFR-NLS mutants did not proliferate in response to EGF or PGE_2_ (Figure 4A *left* and *right* and Supplementary Figure 4A *left* and *right*). Additionally, a clonogenic *in vitro* assay showed that PGE_2_ and EGF increased the number of clones in parental and EGFR WT A549 and GLC82 cells by approximately 50%, whereas in EGFR-NLS mutants cells PGE_2_ or EGF did not promote clonal outgrowth (Figure 4B *left* and *right* and Supplementary Figure 4B *left* and *right)*. Furthermore, qRT-PCR analysis of nuclear EGFR-target genes indicated that PGE_2_ promoted the expression of *CCND1*, *MYC*, *PTGS2* and *NOS2* only in A549 and GLC82 cells bearing EGFR WT, while on the contrary, in EGFR-NLS mutant cells, PGE_2_ did not induce gene expression (Figure 4C and Supplementary Figure 4C).

These results document that PGE_2_ acts as a potent promoter of NSCLC growth and progression by inducing EGFR nuclear translocation and by increasing the expression of nuclear EGFR target genes involved in cell proliferation, cell cycle progression and inflammation.

### PGE_2_ requires EP3 receptor to induce EGFR nuclear translocation

**Figure 5:**
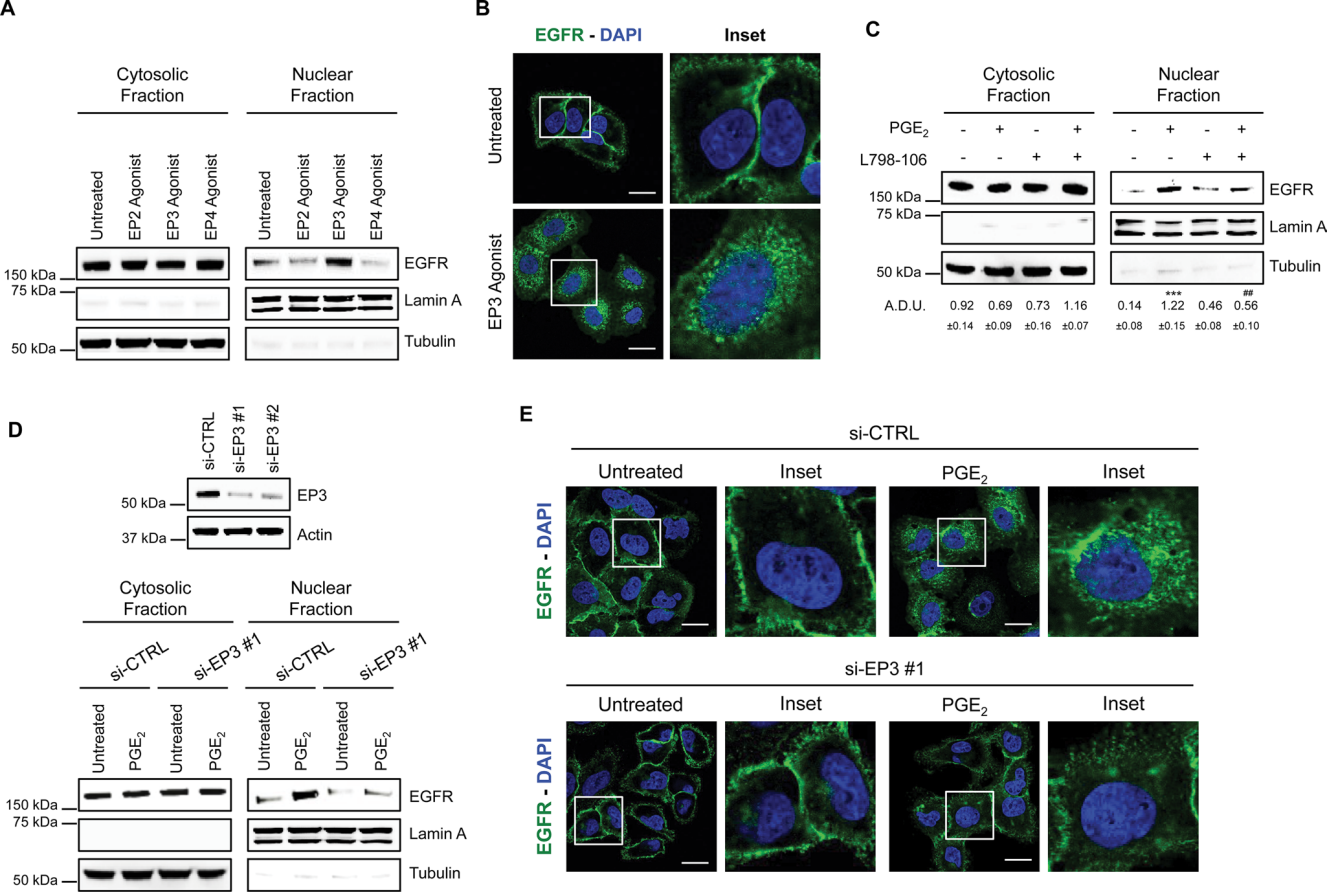
PGE_2_ promotes EGFR nuclear translocation via EP3 receptor (**A**) Immunoblotting analysis of EGFR expression in cytosolic and nuclear fraction in A549 exposed for 60 min to 1 μM EP2, EP3 and EP4 agonists. Tubulin and Lamin A were used as loading control for cytosolic and nuclear fraction, respectively. (**B**) Confocal analysis of EGFR localization in A549 exposed to EP3 agonist for 60 min. EGFR was stained in green, DAPI (blue) was used to counterstain the nuclei. Confocal images were captured in the middle section of the nuclei using 63x objective. Scale bars, 20 μm. Boxed areas are shown in detail in the inset. (**C**) Immunoblotting analysis of EGFR expression in cytosolic and nuclear fraction in A549 cells pretreated with or without EP3 antagonist (L798-106; 1 μM) for 30 min before challenging with 1μM PGE_2_ for 60 min. Immunoblotting quantification was expressed in A.D.U. (arbitrary density unit) and as mean ± SEM. ***p < 0.001 vs Ctrl; ^###^*p* < 0.001 vs PGE_2_. (**D**) A549 cells were transfected with siRNA control or siRNAs against EP3 receptor for 24 h. After that, cells were serum starved overnight and treated with 1 μM PGE_2_ for 60 min. EGFR level in cytoplasmic and nuclear fraction was assessed using western blot with indicated antibodies. Knockdown efficiency was verified by immunoblotting with EP3 antibody, actin was used as loading control. Data are shown only for si-EP3#1, similar data were obtained with si-EP3#2. (**E**) 48 h post transfection, cells were treated with PGE_2_ as indicated in the panels, fixed and stained for EGFR (green) and DAPI (blue). Pictures were acquired in the middle section of nuclei at 63× magnification. Scale bars, 20 μm. Boxed areas are shown in detail in the inset.

To characterize the EP receptor subtype involved in EGFR nuclear translocation, we used specific EP receptor agonists at 1 μM for 60 min: Butaprost as EP2 agonist, Sulprostone as EP3 agonist, and L-902,688 as EP4 agonist. In A549 cells, only the EP3 agonist promoted EGFR internalization indicating its relevance for PGE_2_-mediated EGFR nuclear translocation (Figure 5A). Confocal imaging analysis and 3D reconstruction demonstrated EGFR trafficking and nuclear localization upon EP3 agonist treatment recapitulating PGE_2_ effect (Figure 5B and Supplementary Figure 5). Similar results were obtained in GLC82 cells (Supplementary Figure 6A and 6B). Consistently, the selective antagonist of EP3, L798-106 (10 μM) or siRNA-mediated EP3 silencing (si-EP3) abolished PGE_2_-induced EGFR nuclear translocation, as corroborated by confocal analysis (Figure 5C, 5D, 5E). In si-EP3 cells, EGFR nuclear translocation did not occur upon PGE_2_ treatment and EGFR was confined at the cell membrane as in untreated cells (Figure 5E and Supplementary Figure 7). As a control, EGF-induced EGFR nuclear translocation was not modified in cells with siRNA-ablated EP3 receptor expression (Supplementary Figure 8). These results demonstrate that PGE_2_-mediated EGFR nuclear translocation requires the EP3 receptor.

### EGFR kinase activity is essential for its nuclear translocation

**Figure 6:**
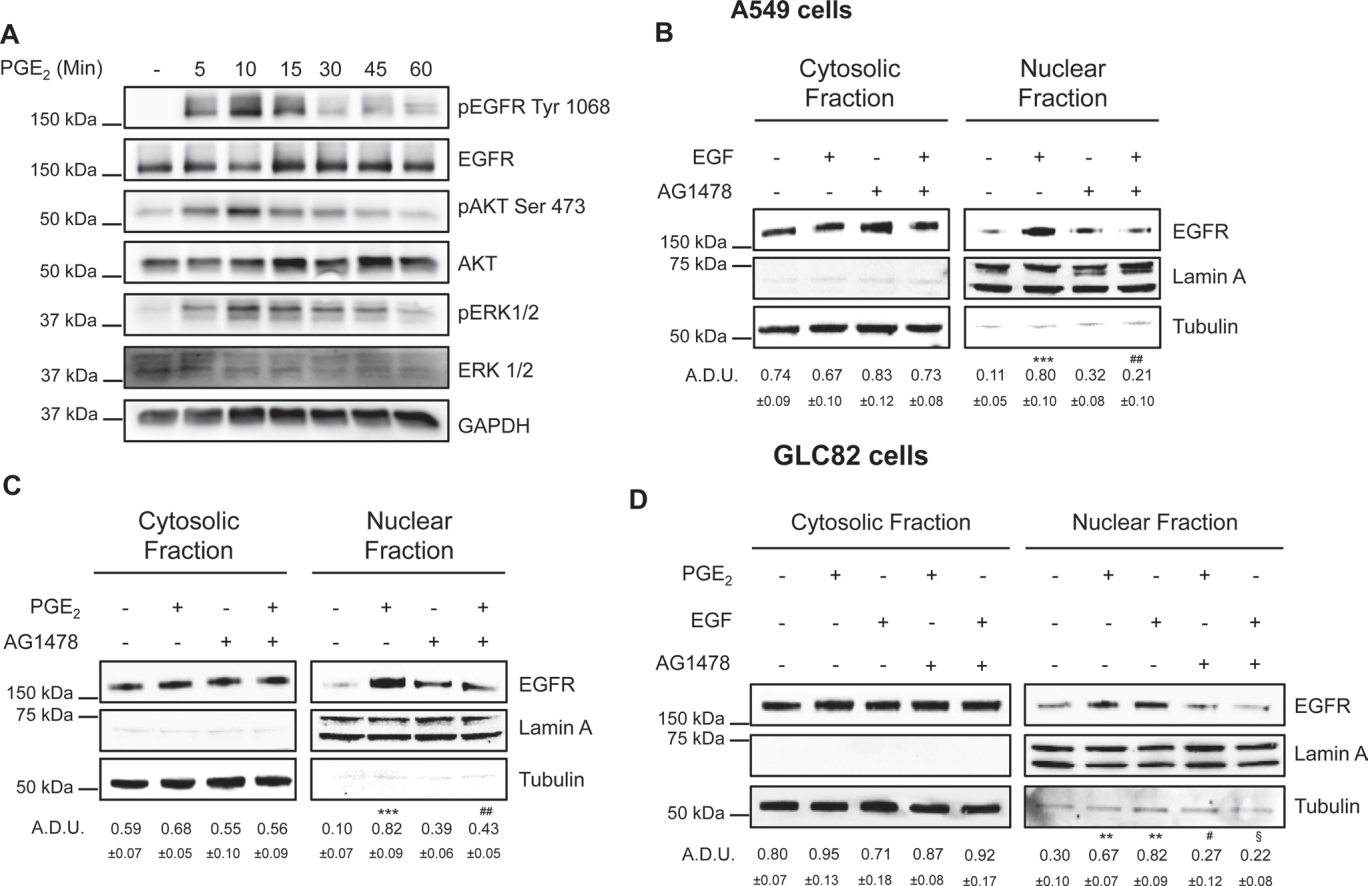
EGFR kinase domain is necessary for its nuclear translocation (**A**) Immunoblotting analysis of EGFR phosphorylation on Tyr1068, and AKT phosphorylation on Ser473 in A549 exposed to 1 μM PGE_2_ for 5–60 min. GAPDH was used as loading control (**B**, **C**) Immunoblotting analysis of EGFR expression in cytosolic and nuclear fraction in A549 exposed for 10 min to 25 ng/ml EGF (B), or 60 min to 1 μM PGE2 (C), with or without pre-incubation with AG1478 (10 μM) for 30 min. (**D**) Immunoblotting analysis of EGFR expression in cytosolic and nuclear fraction in GLC82 exposed for 10 min to 25 ng/ml EGF, or 60 min to 1μM PGE_2_, with or without pre-incubation with 10 μM AG1478 for 30 min. Tubulin and Lamin A were used as loading control for cytosolic and nuclear fraction respectively. Immunoblotting quantification was expressed in A.D.U. (arbitrary density unit) and as mean ± SEM. **p < 0.01, ***p < 0.001 vs Ctrl; ^#^*p* < 0.05, ^##^*p* < 0.01vs PGE_2_; ^§^*p* < 0.05 vs EGF.

To explore whether EGFR nuclear translocation was functionally dependent on its phosphorylation, A549 cells were incubated with PGE_2_ at increasing time points (5–60 min) and EGFR, ERK1/2 and AKT phosphorylation were determined by immunoblotting. EGFR phosphorylation and the downstream signaling pathways were activated in a time-dependent manner with a maximum between 5 and 15 min of PGE_2_ treatment (Figure 6A). We next assessed the requirement of EGFR tyrosine kinase activity for its internalization by incubating NSCLC cells with the EGFR selective tyrosine kinase inhibitor (TKI) AG1478 at 10 μM before exposure to EGF or PGE_2_. AG1478 treatment substantially reduced EGFR nuclear translocation in response to either EGF or PGE_2_ (Figure 6B and 6C), indicating that the tyrosine kinase domain of EGFR is required for nuclear translocation. These results were confirmed in GLC82 cells (Figure 6D).

### PKA, AKT and PKC are not required for PGE_2_-induced nuclear translocation of EGFR

**Figure 7:**
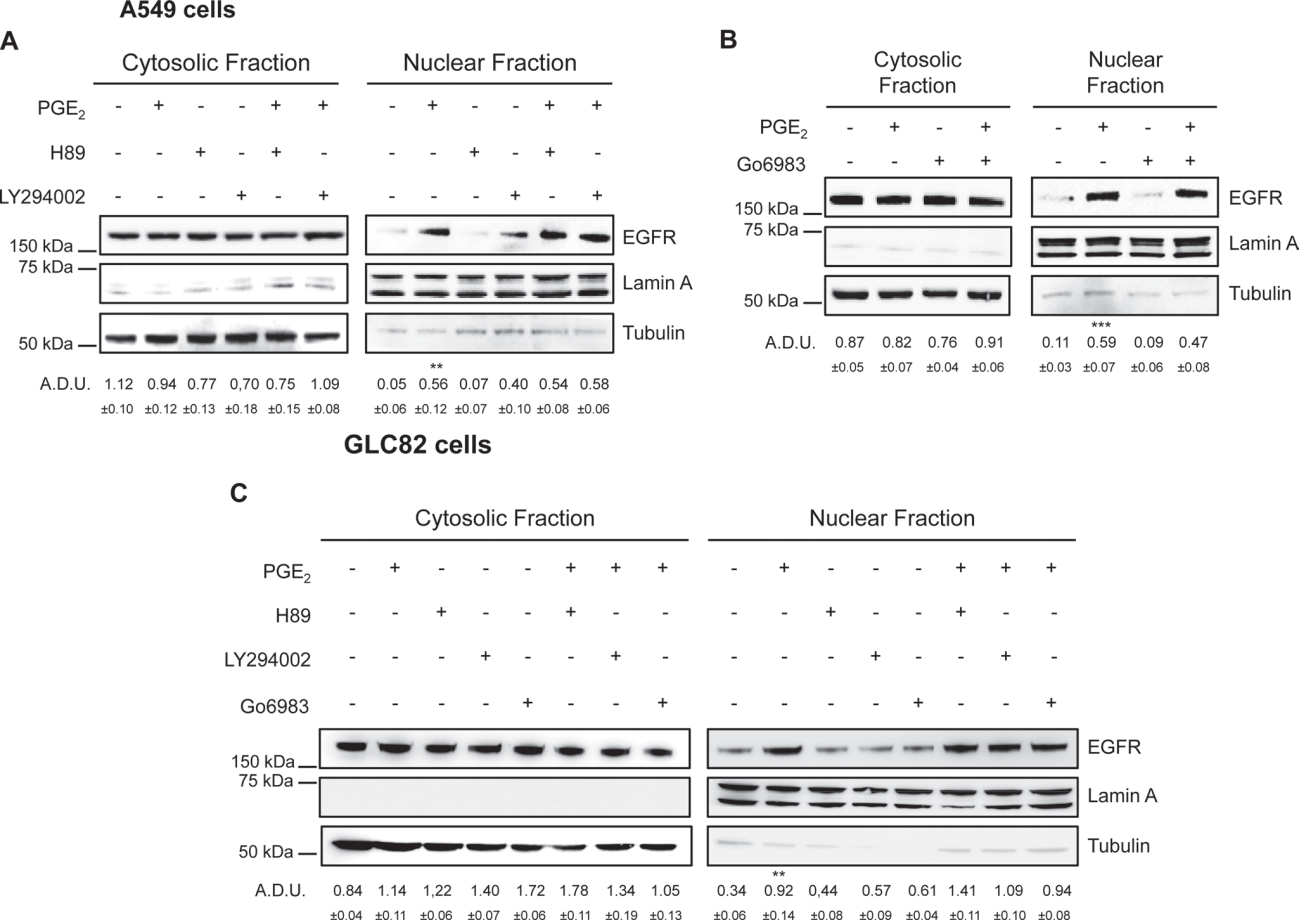
EGFR nuclear translocation in response to PGE_2_ does not involve PKA, AKT and PKC (**A**, **B**, **C**) A549 and GLC82 cells were starved overnight and then treated with 1 μM H89 or 10 μM LY294002 or 10 μM Go6983 for 30 min before challenge with 1 μM PGE_2_ for 60 min. Immunoblotting analysis of EGFR expression on cytoplasmic and nuclear fractions was then performed. Tubulin and Lamin A were used as loading control for cytosolic and nuclear fraction respectively. Immunoblotting quantification was expressed in A.D.U. (arbitrary density unit) and as mean ± SEM. **p < 0.01, ***p < 0.001 vs Ctrl.

To further explore the molecular mechanism, we investigated the functional contribution of potential PGE_2_-EP3 downstream signaling pathways. EP3 receptor consists of multiple isoforms generated by alternative splicing, which upon binding of PGE_2_ trigger different downstream effectors, including protein kinase A (PKA), protein kinase C (PKC), SRC and phosphoinositide 3 kinase (PI3K) known to mediate EGFR activation [33]. To examine which of the protein kinases downstream of EP3 might be critical for EGFR nuclear localization, we assessed the effect of PGE_2_ treatment on EGFR nuclear translocation in the presence of selective inhibitors targeting PKA (H89), PI3K/AKT (LY294002) and PKC (Go6983). None of the selective protein kinases inhibitors affected PGE_2_/EP3-induced EGFR nuclear translocation in A549 (Figure 7A and 7B) and GLC82 cells ( C) excluding functional contribution of PKA, PI3K/AKT and PKC to PGE_2_-mediated EGFR nuclear translocation.

### PGE_2_/EP3 induces EGFR nuclear translocation via SRC family kinases

**Figure 8:**
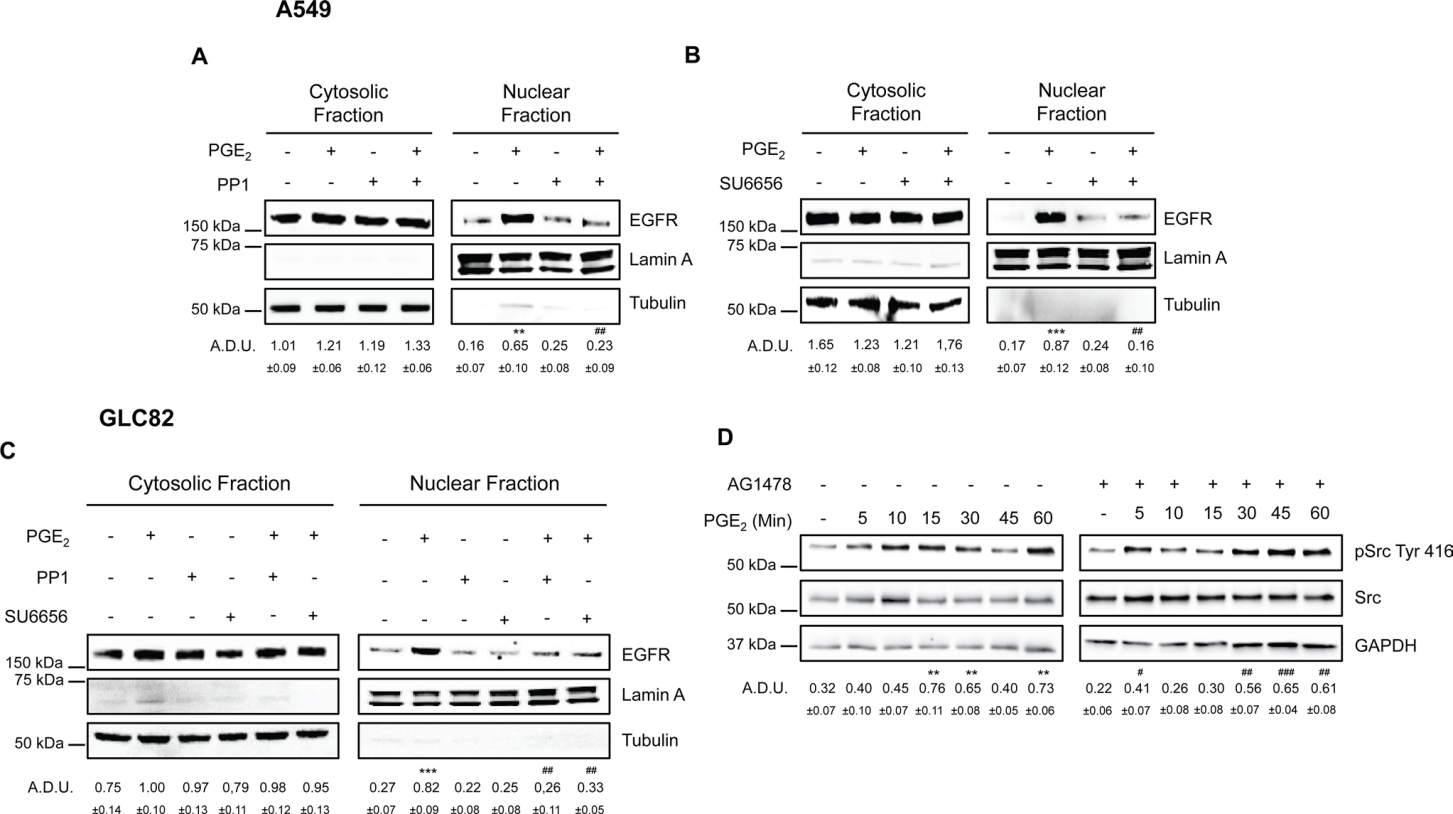
SRC family kinases play a pivotal role in PGE_2_ induced EGFR nuclear translocation Immunoblotting analysis of EGFR expression in cytosolic and nuclear fraction in A549 (**A**, **B**) and GLC82 (**C**) exposed for 60 min to 1 μM PGE_2_ with or without 10 μM PP1 or SU6656. Tubulin and Lamin A were used as loading control for cytosolic and nuclear fraction respectively. Immunoblotting quantification was expressed in A.D.U. (arbitrary density unit) and as mean ± SEM. **p < 0.01, ***p < 0.001 vs Ctrl; ^##^*p* < 0.01 vs PGE_2_. (**D**) Immunoblotting analysis of SRC phosphorylation on Tyr 416 in A549 exposed for 0–60 min to 1 μM PGE_2_ with or without 10 μM AG1478. GAPDH was used as loading control. Immunoblotting quantification of pSRC Tyr 416, normalized to SRC, was expressed in A.D.U. and as mean ± SEM. **p < 0.01 vs Ctrl; ^#^*p* < 0.05, ^##^*p* < 0.01, ^###^*p* < 0.001 vs Ctrl exposed to AG1478.

In addition to the kinases mentioned above, PGE_2_ can also activate SRC Family Kinases (SFK) via EP3 receptor [34, 35]. Notably, this pathway has been shown to serve as a signaling mediator between G protein coupled receptors (GPCRs) and EGFR, as well as downstream effectors of the EGFR [17, 18, 36, 37]. To assess the role of SRC in PGE_2_ induced EGFR nuclear translocation and its relation with EGFR activation, NSCLC cells were treated with PGE_2_ in the presence of pharmacological inhibitors of SRC and EGFR. The SFK inhibitors PP1 or SU6656 abolished PGE_2_-induced EGFR nuclear translocation (Figure 8A, 8B, 8C) whereas did not influence EGF activity (Supplementary Figure 9A and 9B). Inhibition of EGFR activity by AG1478, did not affect PGE_2_-mediated SRC phosphorylation (Figure 8D *left* and *right*). To confirm the central role of SFK in EGFR translocation, a constitutively active SRC (pcSRC-Y527F) was overexpressed in A549 cells. The forced activation of c-SRC, documented by enhanced pSRC phosphorylation, led to an increase in nuclear EGFR localization (Supplementary Figure 9C). Thus, PGE_2_/EP3 signaling acts via SRC to promote EGFR nuclear translocation. However, SRC activation by PGE_2_ does not involve the tyrosine kinase activity of EGFR suggesting that SRC is activated by PGE_2_ upstream of EGFR, subsequently leading to EGFR activation and nuclear localization.

### SRC/ADAMs signaling mediates PGE_2_-induced release of EGFR ligands

**Figure 9:**
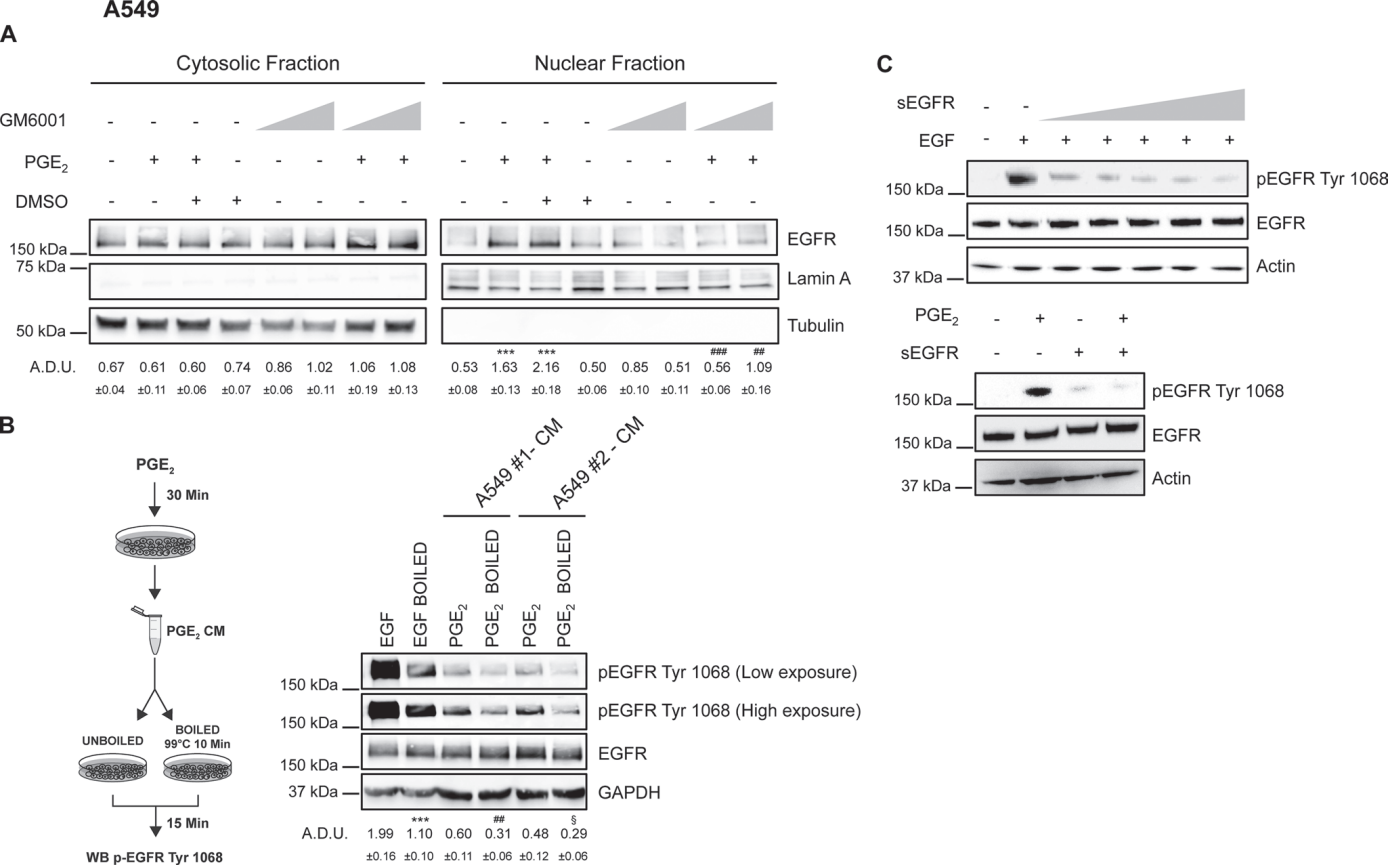
PGE_2_ acts via shedding of EGF-like ligands to promote EGFR nuclear translocation A549 were starved overnight and then pre-treated with 10 μM or 25 μM GM6001 before challenge with 1 μM PGE_2_ for 60 min. DMSO, matching the solvent concentration of 25 μM GM6001, was used as a control. (**A**) Immunoblotting analysis of EGFR expression in cytosolic and nuclear fraction in A549 treated as described above. Tubulin and Lamin A were used as loading control for cytosolic and nuclear fraction respectively. Immunoblotting quantification was expressed in A.D.U. (arbitrary density unit) and as mean ± SEM. ***p < 0.001 vs Ctrl; ^##^*p* < 0.01, ^###^*p* < 0.001 vs PGE_2_+DMSO. (**B**) Overnight starved A549 cells were treated or not with 1 μM PGE_2_ for 30 min. Conditioned medium (CM) was collected from each group and subjected or not to heat inactivation at 99°C for 10 min. Serum-starved A549 cells were stimulated with boiled or unboiled CM for 15 min and then analyzed by immunoblotting of whole cell lysate. Boiled or unboiled medium supplemented with 25ng/ml EGF of 1μM PGE_2_ was used as technical control (data not shown). Immunoblotting analysis of EGFR phosphorylation on Tyr1068 was then performed. A549#1 and A549#2 represent two biological replicates. Low and high exposure were acquired to show protein modulation. GAPDH was used as loading control. Immunoblotting quantification of pEGFR Tyr 1068 (High exposure), normalized to EGFR, was expressed in A.D.U. and as mean ± SEM. ***p < 0.001 vs EGF; ^##^*p* < 0.01 vs PGE_2_ A549#1; ^§^*p* < 0.05 vs. PGE_2_ A549#2. (**C**) A549 cells (left panel) were incubated for 10 min with 5 ng/ml EGF and increasing concentrations of soluble EGFR (sEGFR) (0, 1, 5, 10, 25, 50 μg/ml). Immunoblotting analysis of EGFR phosphorylation on Tyr1068 and total EGFR expression in A549 exposed to 1 μM PGE_2_ and 50μg/ml sEGFR was performed (right panel). Actin was used as loading control.

Since EP-SRC signaling has been reported to activate EGFR by inducing the release of EGFR ligands from the cell membranes [16, 21, 38], we investigated whether PGE_2_ promoted the shedding of EGF-like ligands in NSCLC cells. Cleavage of EGFR ligands is mediated mainly by A disintegrin and metalloproteinases, ADAMs, in particular ADAM10 and ADAM17 represented the major sheddases in mammals [39]. Notably, matrix metalloproteinases (MMPs), such as MMP-2 and MMP-9 are reported to be important regulators in GPCR-induced EGFR ligands shedding [26, 40]. Treatment of A549 or GLC82 cells with the broad-spectrum metalloproteinase inhibitor GM6001 (10 μM or 25 μM) before adding PGE_2_ blocked EGFR nuclear accumulation, indicating that ADAMs-MMPs activation was required for PGE_2_-induced EGFR nuclear translocation (Figure 9A and Supplementary Figure 10A). To explore the contribution of MMP-2, MMP-9, ADAM17 and ADAM10 in mediating the putative PGE_2_-induced EGFR ligands release in NSCLC cells, we assessed their basal expression using qRT-PCR. A549 and GLC82 cells expressed low levels of MMP-2 and MMP-9, whereas ADAM17 and ADAM10 were highly expressed (Supplementary Figure 10B). Additionally, to examine whether PGE_2_ might induces MMPs activation, we performed a gelatin zymography. No lytic activity was observed in both A549 and GLC82 cells exposed to PGE_2_ treatment for 30 min (Supplementary Figure 10C) indicating that ADAMs mediate PGE_2_-induced EGFR ligands cleavage.

Consistent with the hypothesis that EGFR ligands might be released following PGE_2_ treatment by SRC and subsequent ADAMs activation, conditioned medium (CM) of A549 cells treated with PGE_2_ for 30 min was collected and added to untreated A549 to assess EGFR phosphorylation. Putative EGF-like ligands were inactivated in the CM either by heat inactivation and/or by soluble EGFR (sEGFR) as a decoy receptor [19, 41]. CM from two different cell clones, A549#1 and A549#2, was collected and processed for heat inactivation. EGFR phosphorylation was dramatically reduced by heat inactivation of CM suggesting that PGE_2_ activated EGFR by the release of EGF-like ligands in the medium (Figure 9B). Consistently, when sEGFR was added at the maximally effective concentration of 50 μg/ml to A549 and GLC82 exposed to PGE_2_, EGFR phosphorylation was abolished (Figure 9C *left* and *right* and Supplementary Figure 10D). Taken together, these experiments indicate that PGE_2_ activates EGFR by the release of EGF-like ligands in NSCLC cells.

### PGE_2_ requires EGFR ligands to promote EGFR nuclear internalization

**Figure 10:**
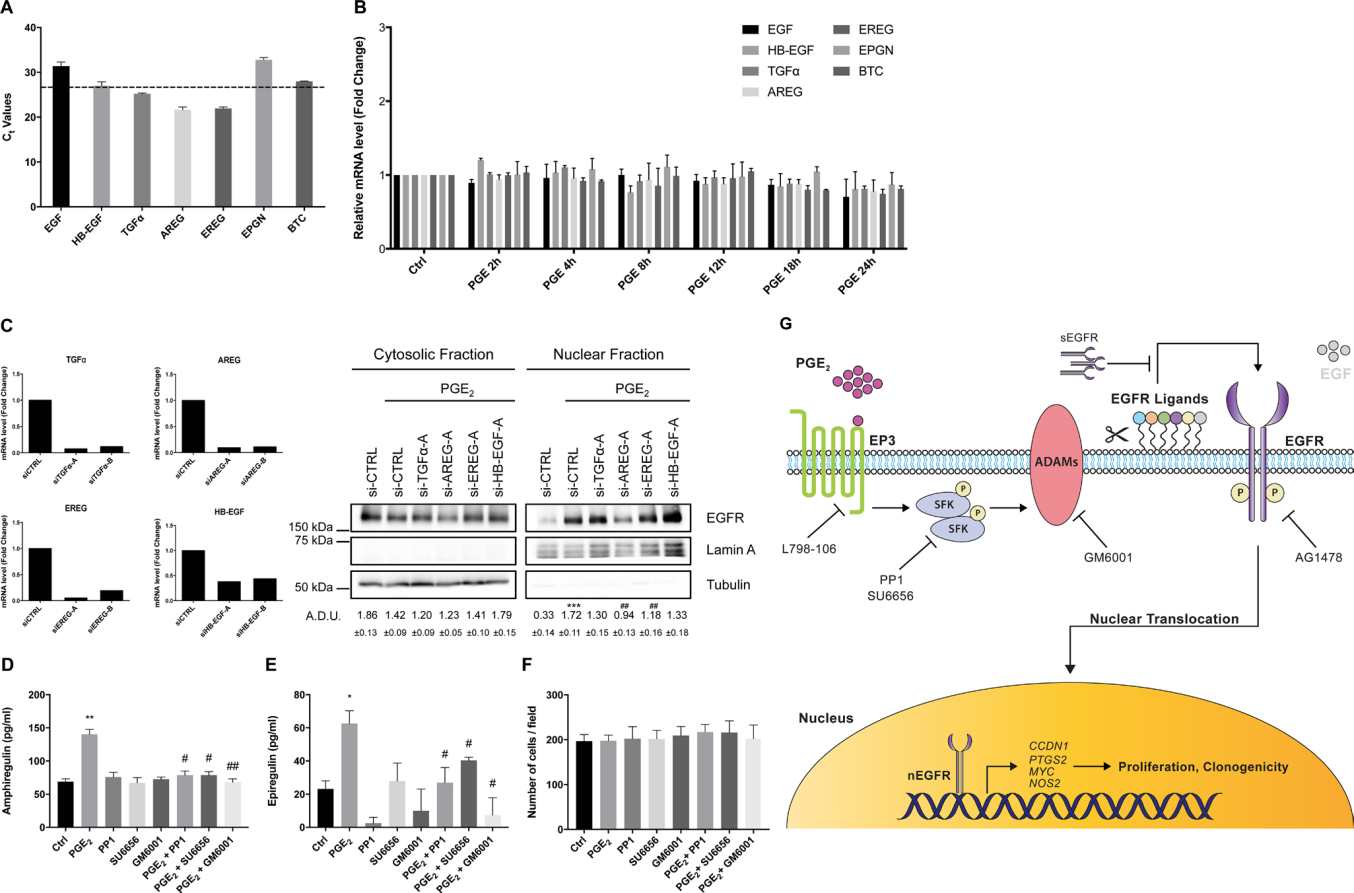
EGFR ligands mediate PGE_2_-dependent EGFR nuclear translocation (**A**) qRT-PCR analysis of basal mRNA expression for EGFR ligands in A549. Results are presented as mean of Ct values ± SEM of two independent experiments. (**B**) A549 cells were starved overnight and then treated with 1 μM PGE_2_ for 2, 4, 8, 12, 18 and 24 h. RNA was isolated and analyzed by qRT-PCR for EGFR ligands. The data are presented as mean of fold change ± SEM of three independent experiments, relative to non-treated cells (Control), which were assigned to 1. (**C**) mRNA expression analysis of EGFR ligands by qRT-PCR in A549 silenced for 48 h for AREG, EREG, TGF-alpha and HB-EGF with two different oligos (siRNA A and B) (left panel). EGFR expression analysis by immunoblotting of cytosolic and nuclear fraction in A549 silenced for EGFR ligands (right panel). Tubulin and Lamin A were used as loading control for cytosolic and nuclear fraction respectively. Immunoblotting quantification was expressed in A.D.U. (arbitrary density unit) and as mean ± SEM. ***p < 0.001 vs siCTRL; ^##^*p* < 0.01 vs siCTRL+PGE_2_. Similar data were obtained with siRNA-B (Data not shown). (**D**, **E**) ELISA for AREG (D) and EREG (E) in conditioned media from A549 exposed for 60 min to PGE_2_ (1 μM), PP1 or SU6656 (10 μM) or GM6001 (10 μM), or PGE_2_ + PP1, PGE_2_ + SU6656 and PGE_2_ + GM6001. Data are reported as pg/ml. *p < 0.05, **p < 0.01 vs Ctrl (control condition); ^#^*p* < 0.05, ^##^*p* < 0.01 vs PGE_2_. (**F**) Cell number in wells exposed to conditions described above for 60 min to obtain the conditioned media. (**G**) Schematic representation of a working model for PGE_2_-induced EGFR nuclear translocation. L798-106, EP3 receptor inhibitor; SFK, SRC family kinases; PP1 and SU6656, SRC family kinases inhibitors; GM6001, broad spectrum ADAM and MMP inhibitor; sEGFR, soluble EGFR; AG1478, EGFR Tyrosine kinase inhibitor; nEGFR, nuclear EGFR.

EGFR is activated by seven ligands, including EGF, HB-EGF, TGFα, AREG, EREG, EPGN and BTC which are all produced as membrane-bound precursor proteins and released by different proteases such as ADAMs [39]. To identify the EGFR ligand(s) involved in PGE_2_-mediated EGFR nuclear translocation, we assessed their basal expression in NSCLC cells using quantitative RT-PCR (qRT-PCR). Both A549 and GLC82 cells expressed variable levels of EGFR ligands (Figure 10A and Supplementary Figure 11A). To assess whether PGE_2_ induced the expression of EGFR ligands, A549 and GLC82 were exposed to the prostanoid for a time frame of 2–24 h, and mRNA levels were determined by qRT-PCR. Upon PGE_2_ treatment, EGFR ligands were not regulated in both NSCLC cells (Figure 10B and Supplementary Figure 11B). The expression of the most expressed ligands for each cell line was individually ablated by siRNA-mediated knockdown, and PGE_2_-dependent EGFR nuclear translocation was assessed. In A549 cells, PGE_2_-induced EGFR nuclear translocation was significantly inhibited by AREG depletion. A reduction of EGFR nuclear accumulation was also observed in EREG silenced cells, whereas in TGFα and HB-EGF knockdown cells, EGFR internalization was marginally affected (Figure 10C), indicating that AREG and EREG were the main ligands mediating PGE_2_-induced EGFR nuclear translocation in A549. Similarly, in GLC82 cells, AREG knockdown significantly decreased PGE_2_-induced EGFR nuclear translocation, although significant reduction of EGFR internalization was also observed in EREG and EGF depleted cells (Supplementary Figure 11C). Taken together these data indicate that several EGFR ligands mediate PGE_2_ activity depending on the cell type, with AREG and EREG being the main involved.

An ELISA assay for AREG and EREG corroborated the contribution of these EGFR ligands to PGE_2_ activity (Figure 10D and 10E, Supplementary Figure 11D and S11E). In tumor cells treated with PGE_2_ for 60 min the levels of AREG and EREG increased, whereas in the presence of c-SRC inhibitors (PP1 or SU6656) or ADAM-MMPs inhibitor (GM6001), AREG and EREG levels declined towards the baseline (Figure 10D and 10E, Supplementary Figure 11D and 11E). This result also supports the notion that c-SRC acts downstream of PGE_2_ to mediate ADAMs activation and EGFR ligands release. Cell numbers were not affected by these treatments (Figure 10F and Supplementary Figure 11F).

In summary, we have identified PGE_2_ as an inducer of EGFR nuclear translocation in human NSCLC cells. PGE_2_ coupling with EP3 receptor orchestrates a complex mechanism involving the activation of SFK and of ADAMs to release EGFR ligands, in particular AREG and EREG. Once activated by its ligands, EGFR translocates to the nucleus where it promotes transcription of genes implicated in cell cycle progression and inflammation leading to increased cell proliferation and clonogenicity (Figure 10G).

## DISCUSSION

The findings presented in this study support a new model for the function of PGE_2_ in tumor growth control and adaptation to the microenvironment, in which the prostanoid regulates EGFR activity by inducing its nuclear internalization. Collectively, our data uncover a key mechanism by which tumor cells attain central hallmarks of cancer by PGE_2_-mediated EGFR nuclear localization.

EGFR is a tyrosine kinase receptor located at the cell surface. In addition to the classical signaling, the full-length EGFR can be shuttled from the plasma membrane to the nucleus in which it serves as co-transcriptional factor and tyrosine kinase [42, 43]. Nuclear EGFR contributes to promote an aggressive phenotype of cancer cells and correlates with poor prognosis and chemo-resistance in different cancer types, including NSCLC [44].

In this report, we identify PGE_2_ as a novel regulator of EGFR nuclear translocation that induces EGFR-mediated tumor cell progression. We delineate the molecular mechanisms and signaling pathways by which PGE_2_ induces EGFR nuclear import and promotes nuclear EGFR-mediated gene transcription in lung adenocarcinoma cells, demonstrating a role for the prostanoid as a critical mediator of EGFR oncogenicity (Figure 10G).

Transcriptional regulation of genes involved in cell proliferation, tumor progression, inflammation and chemo-resistance are among the main functions of nuclear EGFR [43]. We have analyzed a panel of nuclear EGFR target and we find that cyclin D1, COX-2, iNOS and c-Myc mRNA levels are upregulated by PGE_2_ as well as by EGF. The kinetic by which the prostanoid promotes EGFR nuclear translocation and gene transcription appears delayed compared to EGF, suggesting that additional effectors are involved. Among the genes upregulated by EGFR internalization, increased expression of COX-2, the key enzyme in PGE_2_ biosynthesis, indicates a positive feedback loop between PGE_2_/EGFR and COX-2, an instrumental regulatory circuit for the amplification of malignant tumor progression. EGFR nuclear translocation positively correlates with features of tumor aggressiveness: A549 and GLC82 cells expressing wild type EGFR increase their clonogenicity and proliferation in response to PGE_2_ or EGF in contrast to cells expressing a mutant EGFR lacking its nuclear localization sequence.

PGE_2_ exerts its pleiotropic effects by binding to four GPCR (EP1-4) [45]. Using selective EP agonists and antagonists and RNA interference experiments we demonstrate that EP3 is required for PGE_2_-mediated EGFR nuclear translocation in NSCLC cells. This interplay between EP3 and EGFR has been reported previously [46, 47]. In airway epithelial cancer cells, EP3 receptor promotes EGFR-mediated IL-8 production and tumor progression via EGFR ligand shedding [46]. Here, we document that in NSCLC cells PGE_2_-activated EP3 promotes nuclear EGFR translocation by activating SRC family kinases (SFK), which in turn activate ADAMs to cleave and shed EGFR ligands (Figure 10G).

EP3 receptor has multiple isoforms, and its activation can be coupled to adenylyl cyclase [48] and to Ca^2+^-mobilization/PKC activation [49, 50]. EP3 receptor can also stimulate cAMP production leading to PKA activation [51, 52]. In addition PKC [53] and PI3K/AKT [50, 54] are known to act as downstream effectors of EP3 and their contribution to EGFR phosphorylation and nuclear translocation has also been reported [55, 56]. In our setting, PGE_2_/EP3-mediated EGFR trafficking into the nucleus requires EGFR's kinase activity, in contrast to conflicting reports on the importance of an active kinase domain in EGFR nuclear translocation [57–61]. Our experiments reveal that neither PKA, AKT nor PKC are involved in PGE_2_-mediated EGFR nuclear translocation. Conversely, SFK inhibitors markedly impair PGE_2_-mediated EGFR translocation into the nucleus. Thus, we demonstrate that PGE_2_/EP3 acts through SFK to induce EGFR activation and nuclear translocation, a finding consistent with the observation that in NSCLC cells EP3 is functionally connected to SFK [35]. SRC can acts as an upstream or downstream modulator of receptor tyrosine kinases [62]. In our setting, PGE_2_-induced c-SRC phosphorylation appears to be independent of EGFR activation indicating a direct link between SRC and PGE_2_ in promoting EGFR nuclear translocation. The kinetic of cSRC phosphorylation by PGE_2_ was biphasic, as we observed an early peak at 10–15 min and a second delayed peak at 60 min, suggesting that PGE_2_ functions as the initial trigger for a sustained amplification of malignant tumor progression.

PGE_2_ transactivates EGFR by inducing ADAMs-mediated proteolytic release of membrane-bound EGF-like ligands [16, 21, 38], and SFK members play a central role in the release of the ligands [63, 64]. SFK inhibitors block PGE_2_-induced EGFR nuclear translocation, as do ADAM and MMP inhibitors, suggesting that EGF-like ligands are shedded in NSCLC cells treated with PGE_2_. A dramatic reduction in the extent of EGFR phosphorylation occurs in cells, when conditioned medium from NSCLC cells is denatured or depleted of EGFR ligands with a soluble EGFR trap, demonstrating that PGE_2_ promotes EGFR activation and internalization through cleavage of membrane-bound EGFR ligands. Among the various EGFR ligands, we demonstrate that the shedding of Amphiregulin (AREG) and Epiregulin (EREG) play a central role in EGFR trafficking. Both ligands are known oncogenic factor [65–68]. In advanced NSCLC patients, increased AREG expression correlates with a poor response to therapy, and several studies have identified AREG as a biomarker for an efficient response to EGFR-targeted therapies [69–71]. Further, PGE_2_ has been reported to promote AREG induction and to stimulate growth of colon cancer cells [72]. Similarly, elevated EREG expression in NSCLC is associated with aggressive tumor phenotypes and unfavourable prognosis [73–75]. Additionally, in several tumor cell lines, COX-2 and EREG have been identified as metastasis associated genes [76, 77].

In summary, we have identified PGE_2_ as an inducer of EGFR nuclear translocation in human NSCLC cells. We propose the following mechanistic sequence of events: tumor stroma and/or tumor cells release PGE_2_, which couples with EP3 receptor and orchestrates a complex mechanism culminating in EGFR activation and translocation into the nucleus. We show that upon PGE_2_/EP3 interaction, SFKs activate ADAMs proteases, which in turn mediate the shedding of EGFR ligands, such as the oncogenic AREG and EREG, and then EGFR activation and nuclear internalization. Within the nucleus, EGFR induces the expression of iNOS, COX-2, c-Myc and cyclin D1, thus reprogramming important tumor growth parameters, including tumor cell proliferation and malignant progression (Figure 10G). The delayed gene transcription, observed in NSCLC cells exposed to PGE_2_, represents a clear functional evidence of the involvement of the release of EGFR ligands in this mechanism. Thus, it appears that, through EGFR nuclear translocation, PGE_2_ was able to amplify a robust oncogenic response, sustained by a wide variety of inflammatory and pro-proliferative genes.

The presence of receptor tyrosine kinase into the nucleus opens a new field of research. Here we demonstrate the significance of the nuclear translocation induced by PGE_2_-mediated GPCR signaling and its biological functions. These mechanisms regulating tumor growth and malignant progression may offer attractive opportunities for the design and development of innovative cancer therapies.

## MATERIALS AND METHODS

### Cell culture and cultured conditions

The human NSCLC cancer cell line A549 (CCL-185), was obtained from American Type Culture Collection and the GLC82 NSCLC cell line was kindly provided by Dr. Mario Chiariello (Istituto Toscano Tumori, Siena, Italy). Cells were certified by STRA, (LGC Standards S.r.l., Sesto San Giovanni, Milan, Italy) and were maintained in DMEM for A549 and in RPMI-1640 (Euroclone, Milan, Italy) for GLC82 supplemented with 10% FBS and 2 mM Glutamine, 100 Units Penicillin and 0.1 mg/l Streptomycin (Sigma Aldrich, St. Louis, MO, USA) in a humidified incubator with 5% CO2 at 37°C. A549 and GLC82 were immediately expanded after delivery (up to 6 × 10^7^ cells) and frozen down (1 × 10^6^ per vial) such that both cell lines could be restarted after a maximum of 10 passages every 3 months from a frozen vial of the same batch of cells. Control of mycoplasma was done from a frozen vial.

### Chemical and reagents

Recombinant human EGF and soluble EGFR were purchased from PeproTech (Rocky Hill, NJ, USA). PGE_2_, L-798106, PP1 and SU6656 were purchased from Sigma Aldrich. Butaprost (EP2 agonist), Sulprostone (EP3 agonist), L-902,688 (EP4 agonist), Tyrphostin AG-1478 and GM6001 were obtained from Cayman Chemicals (Ann Arbor, MI, USA). H89 and LY294002 were purchased from Calbiochem (Darmstadt, Germany). Go6983 was obtained from Tocris (Bristol, United Kingdom).

### Antibodies

Anti-EGFR, anti-pEGFR Tyr 1068, anti-AKT, anti-pAKT Ser 473, anti-ERK1/2, anti-pERK1/2, anti-SRC, anti-pSRC Tyr 416 antibodies were purchased from Cell Signaling Technology (Danvers, MA, USA). Anti-Tubulin and anti-EP3 receptor antibodies were purchased from Santa Cruz (Heidelberg, Germany). Anti-Lamin A, anti-Actin and anti-GAPDH antibodies were obtained from Sigma Aldrich. Anti-EGFR (N-terminal) was purchased from Abcam (Cambridge, United Kingdom).

### Whole cell extracts

Cells were washed 2× with cold Dulbecco's Phosphate Buffered Saline (Sigma-Aldrich) and lysed as described previously [78].

### Cell fractionation

Nuclear and cytoplasmic extracts were prepared with NE-PER^™^ nuclear and cytoplasmic extraction reagents (ThermoFisher Scientific, Waltham, MA, USA) following the manufacturer's instructions.

### Immunoblotting analysis

4 × 10^5^ cells were plated in 60 mm dishes, serum deprived (0.1%. fetal calf serum, overnight), then treated as described in the text. Immunoblot analysis was performed as described previously [17]. Signals were detected by SuperSignal WestPico Chemiluminescent Substrate (ThermoFisher Scientific) using ChemiDoc system and Quantity one software (Bio-Rad, Hercules, CA, USA). All experiments were performed at least three times. For all experiments using whole cell lysate, GAPDH or Actin were used as loading control. Lamin-A and Tubulin were used as loading and purity controls for the nuclear and cytosolic fractions, respectively. Immunoblots were analyzed by densitometry using NIH Image J 1.48v software, and the results, expressed as arbitrary density units (A.D.U.), were normalized to GAPDH, Actin, Lamin-A or Tubulin.

### Immunofluorescence microscopy analysis

Cells were plated on 12 mm ø glass coverslips, starved overnight and treated according to the experimental design. Cells were fixed and incubated with anti-EGFR antibody followed by AlexaFluor^®^ 488-labeled secondary antibody (Invitrogen, Carlsbad, CA, USA) as previously described [78]. 6-diamidino-2- phenylindole (DAPI) 1 μg/ml (Sigma-Aldrich) was used to stain the nuclei. Cells were imaged with a confocal laser scanning microscope Leica SP5. Images for documenting EGFR nuclear translocation were acquired in the middle section of the nuclei with 63× magnification. Confocal stacks were 3D-reconstructed with Imaris Software (Bitplane, Zurich, Switzerland).

### Transfection of siRNAs and plasmids

siRNAs used for transient knock-down experiments were purchased from Qiagen (Hilden, Germany) and Ambion (Carlsbad, CA, USA). Cells were transfected with 20 nM targeting siRNA or scrambled control siRNA using Lipofectamine^®^ RNAiMAX (Invitrogen) according to manufacturer's instructions. Cells were assayed 48–72 h after transfection. Knockdown efficiency was assessed by immunoblotting or quantitative RT-PCR analysis. Target sequences are listed in Supplementary Table 1.

For DNA transfection, cells were transfected with 1–10 μg plasmid using Lipofectamine^®^ 2000 (Invitrogen) according to manufacturer's instructions. EGFR WT and NLS mutant plasmids (NLSm12 and dNLS) were kindly provided by Prof. Mien-Chie Hung (University of Texas MD Anderson Cancer Center, Houston, TX, USA) [32]. pSpCas9(BB)-2A-GFP (PX458) (#48138) pEVX (#17675), and pSRCY527F (#17675) were from Addgene. Cells were analyzed 24–72 h post-transfection.

### Knockout of EGFR by CRISPR/Cas9-mediated genome editing

A549 EGFR knockout cells were generated by a CRISPR/Cas9 approach as described [79]. The sgRNA with the sequence TCGTTCGGAAGCGCACGCTGCGG within the EGFR gene was obtained using the CRISPR Design Tool (http://tools.genome-engineering.org). sgRNA targeting EGFR was cloned into BbsI (NEB, Ipswich, MA, USA) digested pSpCas9(BB)-2A-GFP (PX458) plasmid (Addgene #48138) using the oligos: Forward CACCGTCGTTCGGAAGCGCACGCTG and Reverse AAACCAGCGTGCGCTTCCGAACGAC. Cells were transiently transfected with 1 μg PX458 using an Amaxa Nucleofector machine (Lonza, Basel, Switzerland) according to manufacturer's instructions. 48 h post transfection, GFP-expressing cells were FACS sorted and re-seeded at limiting dilution in 96-well plates in order to obtain individual clones. GLC82 EGFR knockout cells were generated using CRISPR/Cas9 human gene knockout kit (OriGene, Rockville, MD, USA) following manufacturer's instructions. EGFR ablation was assessed by immunoblot with two different anti-EGFR antibodies targeting C- and N-terminal residues respectively.

### RNA isolation and quantitative RT-PCR

Total RNA was prepared using Tri Reagent^®^ (Sigma-Aldrich) following manufacturer's instructions. 1 μg RNA was reverse transcribed using ImProm- II^™^ Reverse Transcriptase (Promega, Madison, WI, USA) and quantitative RT-PCR was performed using SYBR-green PCR MasterMix (Applied Biosystems, Waltham, MA, USA) in a StepOne Plus PCR machine (Applied Biosystems). Fold change expression was determined by the comparative Ct method (ΔΔCt) normalized to 60S Ribosomal protein L19 expression. qRT-PCR data are represented as fold increase relative to non-treated cells (Control), which were assigned to 1. Primers for quantitative RT-PCR are listed in Supplementary Table 2.

### Conditioned medium and EGFR ligands denaturation

5 × 10^5^ A549 cells were plated into 60 mm dishes, incubated for 24 h and then starved overnight with medium supplemented with 0.1% FBS. Then, cells were incubated with or without 1 μM PGE_2_ for 30 min in a total volume of 4 ml. Conditioned medium (CM) was collected and boiled or not at 99°C for 10 min. As control for EGFR ligands denaturation efficiency, boiled or unboiled medium supplemented with EGF 25 ng/ml was used. Next, media of serum-starved A549 cells were replaced with either 2 ml of boiled or unboiled CM or with 2 ml of boiled or unboiled medium derived from controls. Cells were treated for 15 min and subsequently analyzed by immunoblotting.

### Gelatin zymography

5 × 10^3^ A549 and GLC82 cells were plated into 96-well plates in medium supplemented with 10% FBS. After adhesion, cells were washed with PBS and starved overnight in serum-free medium. 1 μM PGE_2_ was added to 50 μL of fresh serum-free media for 30 min. Next, conditioned medium (CM) was collected and mixed with loading buffer. Zymography was carried out in SDS/8% PAGE containing 0.1% gelatin as described [31].

### ELISA

1 × 10^5^ A549 and GLC82 cells were plated into 12-well plates and incubated until 80–90% confluency. Then, cells were starved overnight and treated as described in the text. CM was collected and Amphiregulin and Epiregulin levels were measured using an ELISA kit R&D Systems, Minneapolis, MN, USA for Amphiregulin and an ELISA kit MyBioSource, San Diego, CA, USA for Epiregulin, following the manufacturer's instructions.

### MTT assay

Cell proliferation was quantified by the Vybrant MTT cell proliferation assay as previously described [17]. Briefly, A549 and GLC82 EGFR knockout cells were transfected with EGFR WT and NLS mutant plasmids for 24 h. Next, transfected cells were seeded (3 × 10^3^) into 96-well plates, starved overnight and treated with either 25 ng/ml EGF or 1 μM PGE_2_. 48 h post treatment, cell were exposed to MTT (3-(4,5-dimethylthiazol-2-yl)-2,5-diphenyltetrazolium bromide (Sigma-Aldrich) for 4 h in fresh medium without phenol red. Absorbance at 540 nm was measured with Infinite 200 Pro SpectraFluor microplate absorbance reader (Tecan, Mannedorf, Switzerland). Data of three independent experiments are presented as % relative to untreated cells (Control), which were assigned to 100%.

### Clonogenic assay

A549 and GLC82 EGFR knockout cells were transfected with EGFR WT and NLS mutant plasmids for 24 h. Transfected cells were seeded (5 × 10^2^) into 6-well plates and incubated in medium supplemented with 10% FBS for 12 h. Then, cells were treated in triplicates with 25 ng/ml EGF or 1 μM PGE_2_ in 1% FBS medium. 10 days after treatment, cells were stained with Panreac kit (Darmstadt, Germany), and colonies (> 50 cells) were counted; data are expressed as % relative to untreated cells (Control), which were assigned to 100%.

### Statistical analysis

Statistical analysis and graphs were generated using the GraphPad Prism software (San Diego, CA, USA). All statistical analysis was done by unpaired/paired Student's *t*-test, *p*-value < 0.05 was considered significant.

## SUPPLEMENTARY MATERIALS FIGURES AND TABLES


