# Supplementary material for: PGE2/EP3/SRC signaling induces EGFR nuclear translocation and growth through EGFR ligands release in lung adenocarcinoma cells
Source: Oncotarget. 2017 Mar 10;8(19):31270–87. doi: 10.18632/oncotarget.16116 (PMC5458206; doi:10.18632/oncotarget.16116)
Supplement: Supplementary file 1 [file oncotarget-08-31270-s001.pdf]

PGE_2/EP3/SRC signaling induces EGFR nuclear translocation and growth through EGFR ligands release in lung adenocarcinoma cells

Supplementary Materials

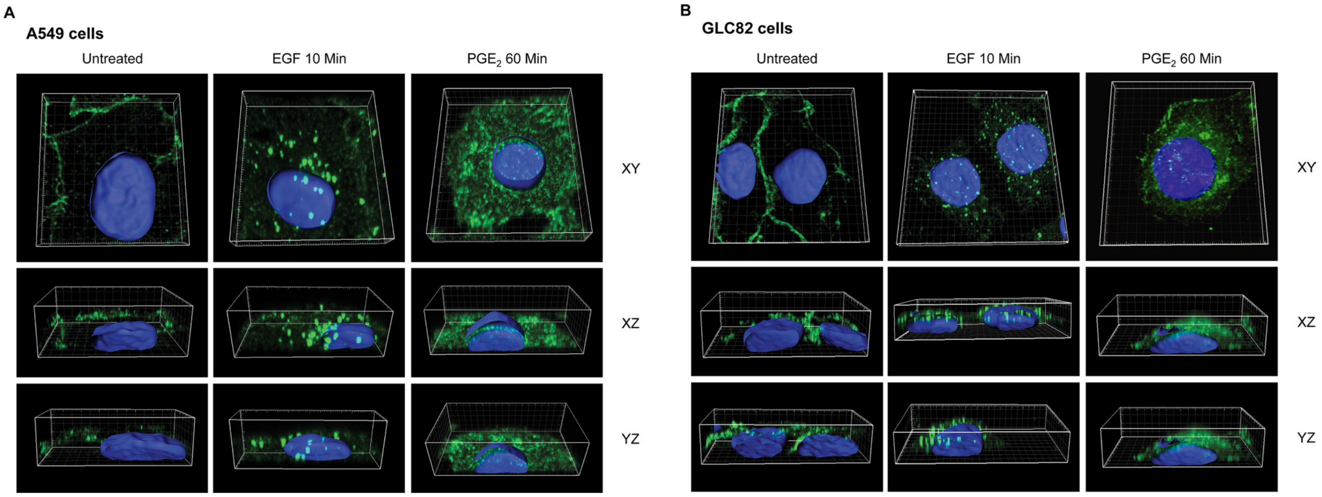

Supplementary Figure 1: Three-dimensional reconstruction of confocal imaging stacks from A549 and GLC82 cells treated with EGF or PGE_2. 3D reconstruction of confocal stacks showing EGFR localization in A549 (**A**) and GLC82 (**B**) cells. Untreated cells (left panels) or exposed to 25 ng/ml EGF (10 min, middle panels) or 1 µM PGE_2 (60 min, right panels) were reconstructed in XY, XZ and YZ axis. EGFR is visualized in green and the nuclei in blue.

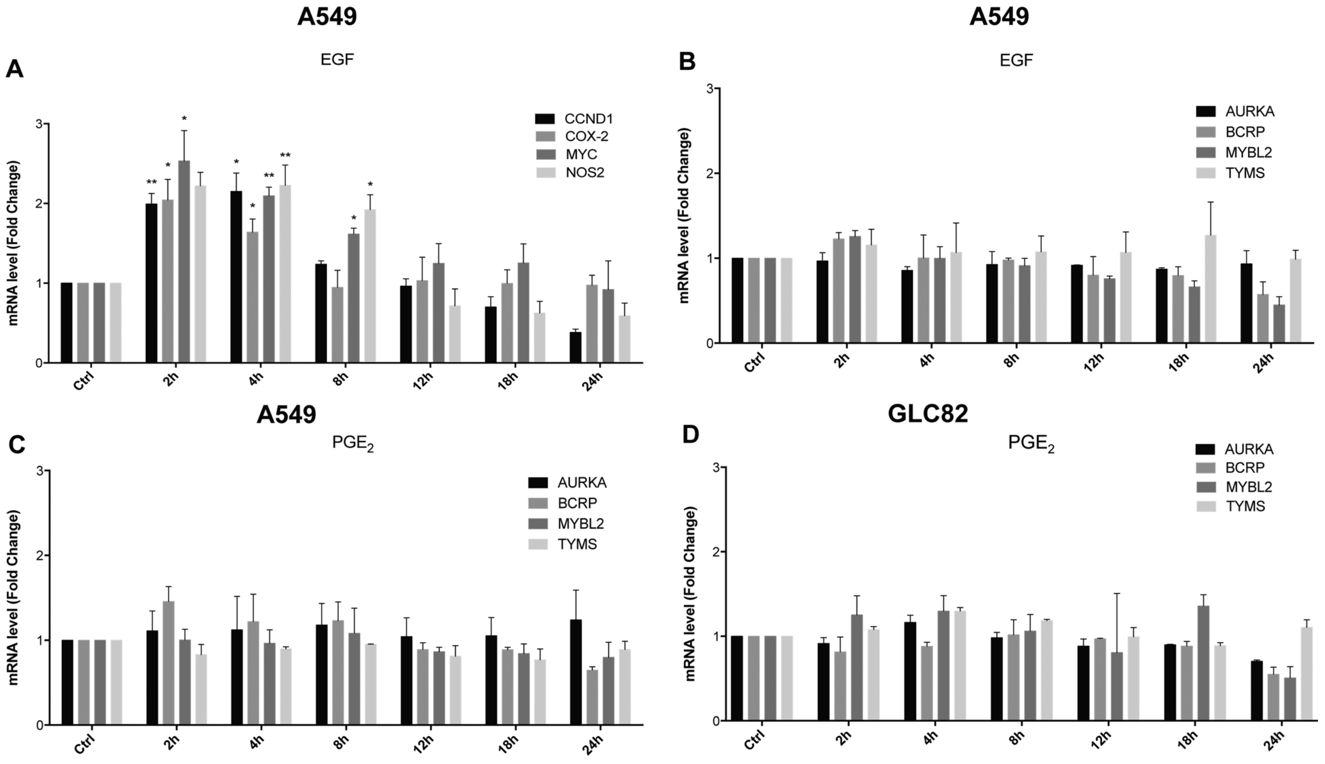

Supplementary Figure 2: Nuclear EGFR target genes regulated or not by EGF or PGE_2. (**A**) A549 cells were starved overnight and then treated with 25ng/ml EGF for 2-24 h. RNA was isolated and analyzed by qRT-PCR for a panel of nuclear EGFR target genes. (**B**, **C**, **D**) qRT-PCR analysis for mRNA expression of EGFR targeted genes (AURKA, BCRP, MYBL2 and TYMS) in A549 exposed for 2-24 h to 25 ng/ml EGF (B) or 1 µM PGE_2 (C) and GLC82 exposed for 2-24 h to 1 µM PGE2 (D). qRT-PCR data are presented as mean of fold change ± SEM of three independent experiments, relative to non-treated cells (Control), which were assigned to 1.

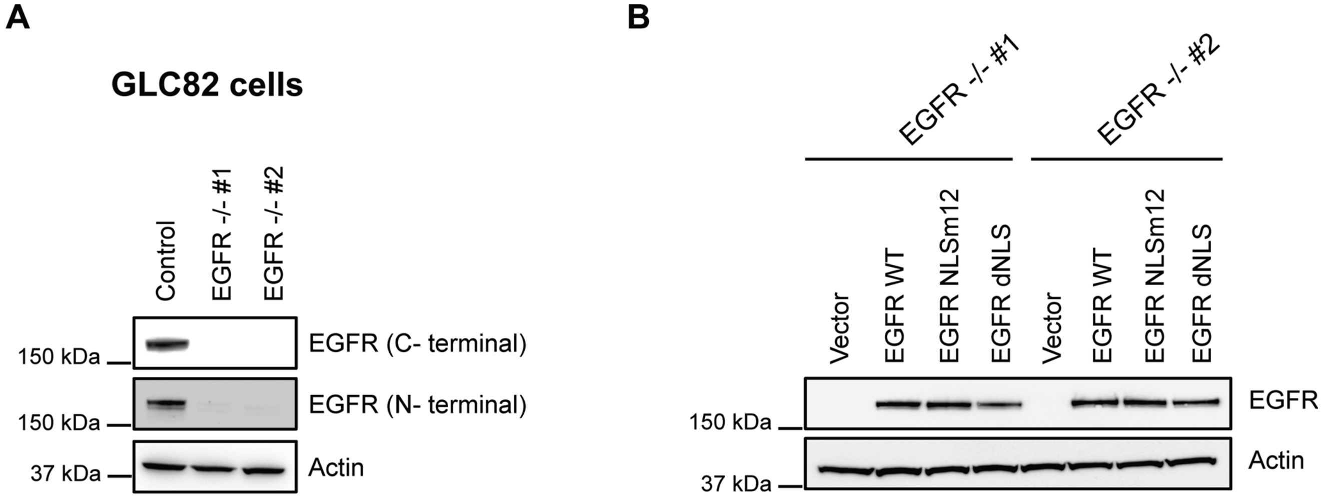

Supplementary Figure 3: NSCLC cell models to study PGE_2-induced EGFR nuclear translocation. (**A**) Immunoblotting analysis of EGFR expression in GLC82 wild type cells and two clones knockout for EGFR, generated by CRISPR/Cas9 (EGFR −/− #1, #2). Actin was used as loading control. (**B**) Expression of EGFR in EGFR −/− #1, #2 GLC82 cells transfected with Vector, EGFR-WT and NLS mutant plasmids for 72 h.

GLC82 cells

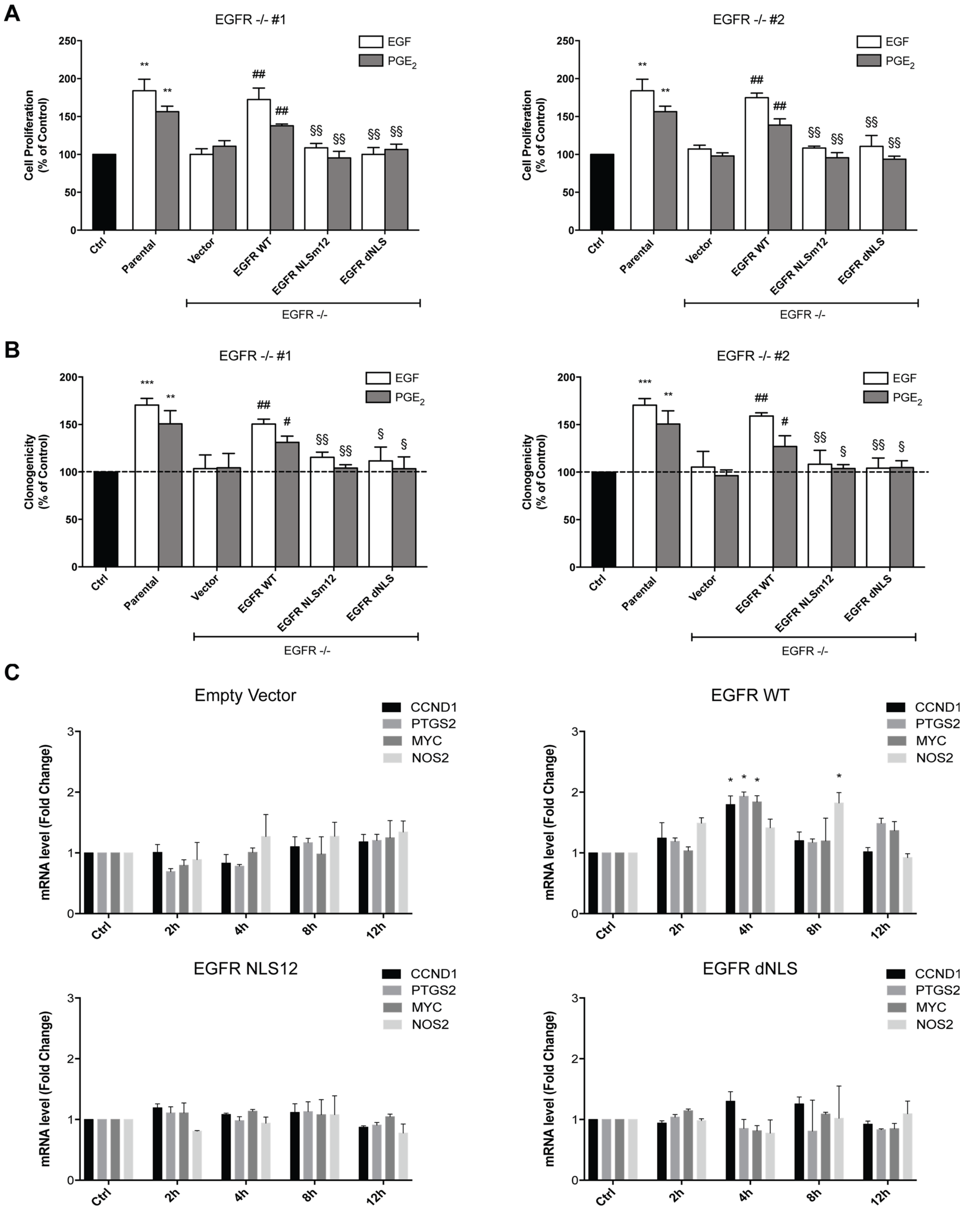

Supplementary Figure 4: PGE_2 promotes cell proliferation, clonogenicity and gene regulation via nuclear EGFR in GLC82. (**A**, **B**, **C**) Parental GLC82 cells or EGFR −/− #1, #2 cells transfected with Vector or EGFR-WT or NLS mutant plasmids were seeded and incubated for 24 h. Next, cells were harvested and seeded for MTT, clonogenic assay and RNA isolation. (A) Cell growth was assessed by MTT assay after 48 h treatment with 25 ng/ml EGF or 1μM PGE2. Data are presented as mean ± SEM of triplicate cultures, expressed as % of control. $^{**}p < 0.01$ vs Ctrl; $^{\#\#}p < 0.01$ vs Vector; $^{\S\S}p < 0.01$ vs EGFR WT. (B) Clonal outgrowth was assessed by counting number of clones (> 50 cells) 12 days after treatment with 25 ng/ml EGF or 1 μM PGE_2. Data are presented as mean ± SEM of triplicates, expressed as % of control. $^{**}p < 0.01$, $^{***}p < 0.001$ vs Ctrl; $^{\#}p < 0.05$, $^{\#\#}p < 0.01$ vs Vector; $^{\S}p < 0.05$, $^{\S\S}p < 0.01$ vs EGFR WT. (C) RNA was isolated after 2, 4, 8, 12 h treatment with 1μM PGE2 and analyzed by qRT-PCR for regulated nuclear EGFR target genes. The data are presented as fold change ± SEM of three independent experiments, relative to non-treated cells (Control), which were assigned to 1. $^{*}p < 0.05$ vs Ctrl.

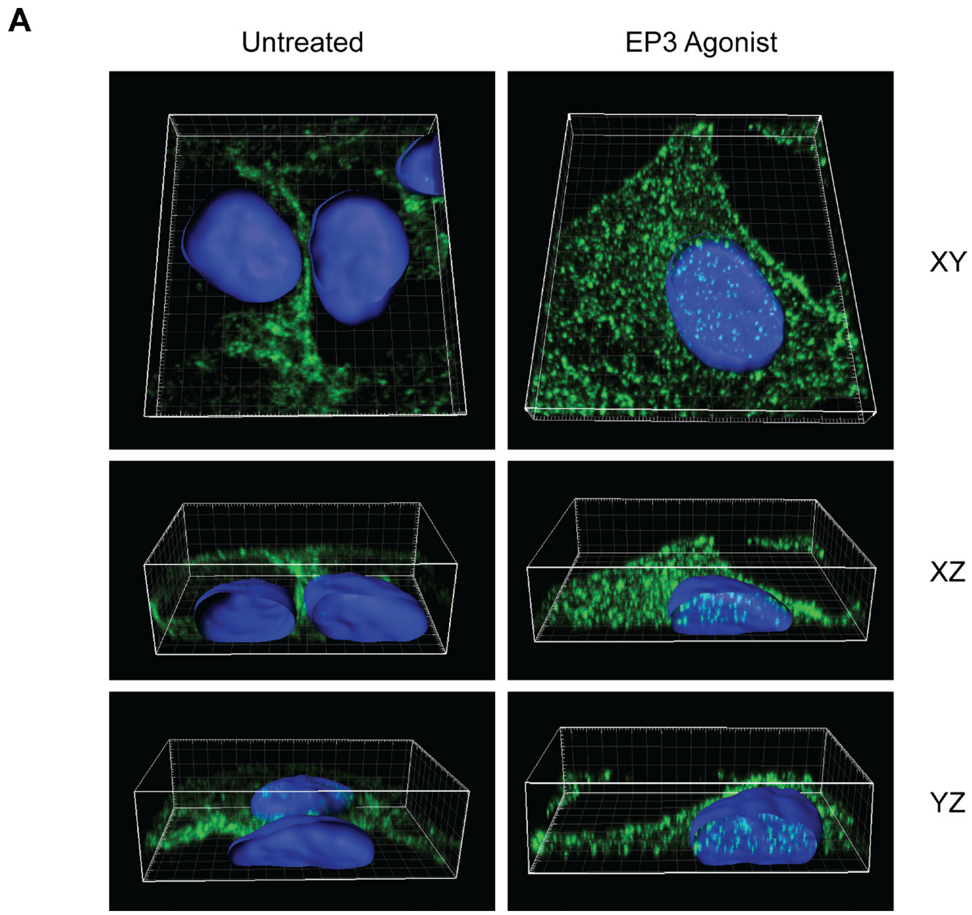

Supplementary Figure 5: Three-dimensional reconstruction of A549 cells showing EGFR nuclear translocation upon EP3 agonist treatment. 3D reconstruction of confocal stacks showing EGFR localization in untreated A549 cells (left panels) or exposed to the EP3 agonist Sulprostone at 1 µM for 60 min (right panels). Confocal images were reconstructed in XY, XZ and YZ axis. EGFR is visualized in green and the nuclei in blue.

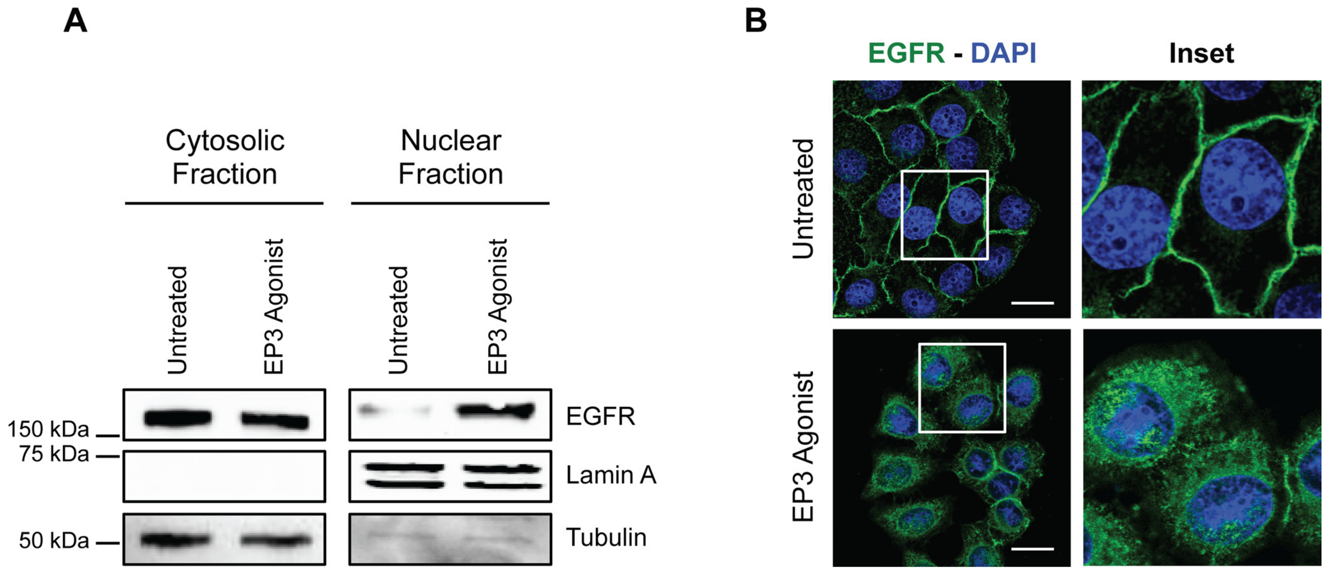

Supplementary Figure 6: EP3 receptor mediates PGE_2-induced EGFR nuclear translocation in GLC82. (**A**) Immunoblotting analysis of EGFR expression in cytosolic and nuclear fraction in GLC82 cells exposed for 60 min to 1 µM EP3 agonist. (**B**) Confocal analysis of EGFR localization in GLC82 treated with EP3 agonist for 60 min. EGFR was stained in green, DAPI (blue) was used to counterstain the nuclei. Confocal pictures were acquired in the middle section of nuclei at 63× magnification. Scale bars, 20 µm. Boxed areas are shown in detail in the inset.

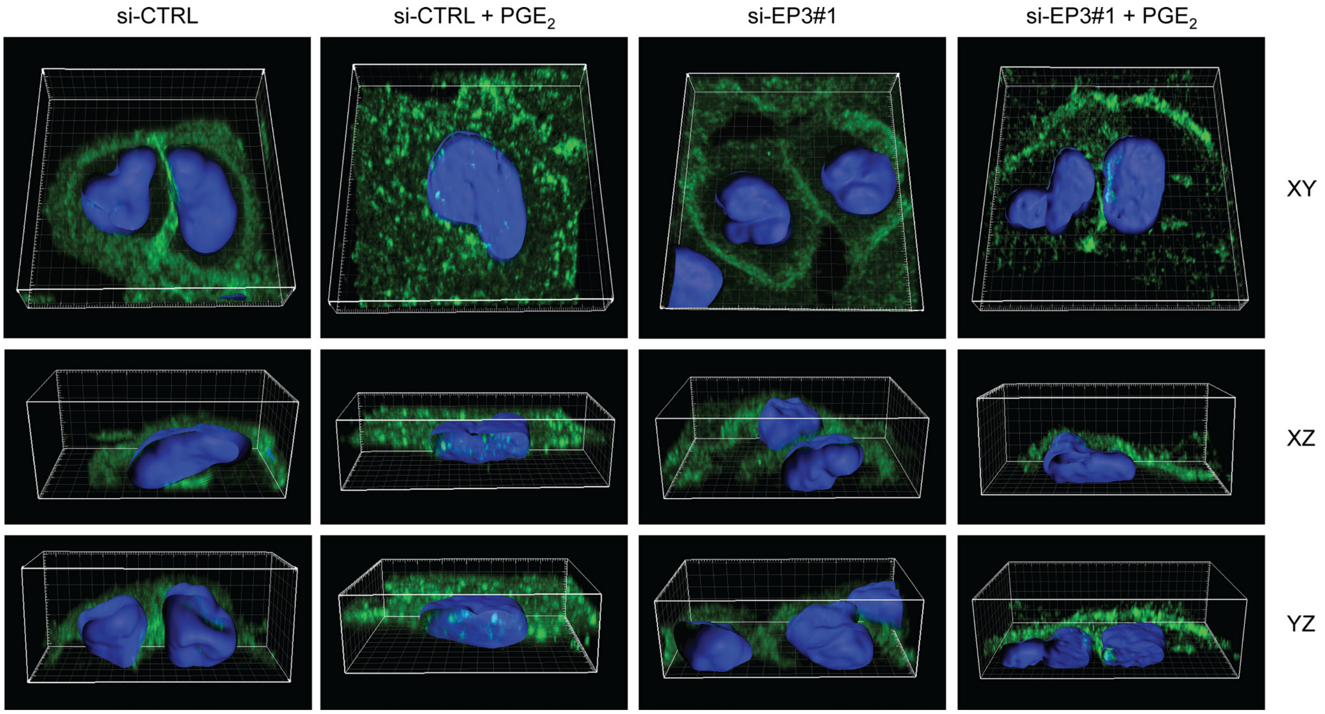

Supplementary Figure 7: Three-dimensional reconstruction of confocal imaging stacks of EP3-silenced A549 cells. 3D reconstruction of confocal stacks showing EGFR localization in A549 cells transfected with siRNA control or siRNAs against EP3 receptor. 48 h post transfection, cells were treated or not with 1 μM PGE_2 for 60 min as indicated in the panels. Images were reconstructed in XY, XZ and YZ axis. EGFR is visualized in green and the nuclei in blue.

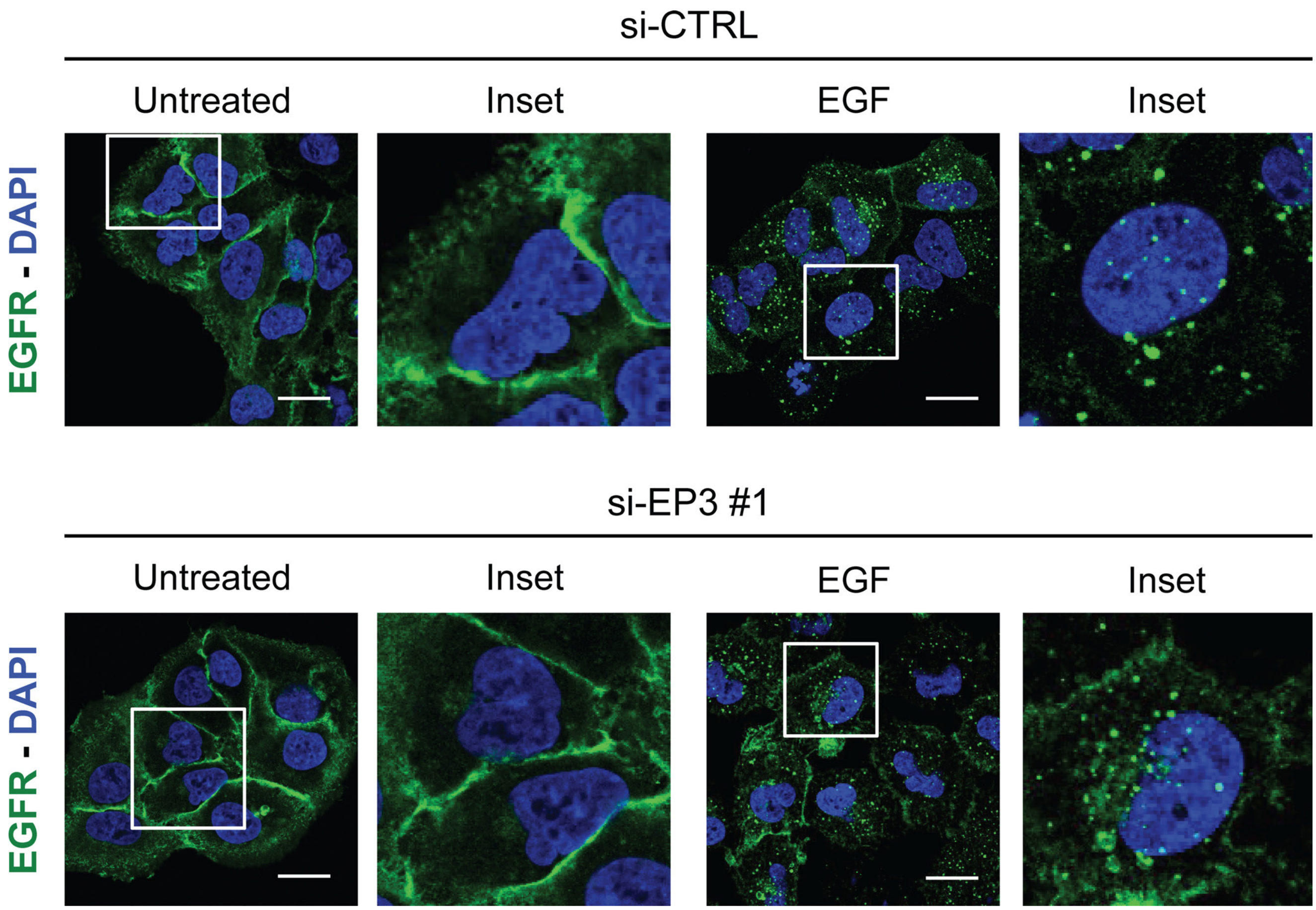

Supplementary Figure 8: EP3 knockdown does not alter EGF-induced EGFR nuclear translocation. A549 cells were transfected with siRNA control or siRNAs against EP3 receptor for 24 h. After, cells were starved overnight and treated with 25 ng/ml EGF for 10 min. Cells were stained for EGFR (green) and DAPI (blue). Confocal pictures were acquired in the middle section of nuclei at 63× magnification. Scale bars, 20 µm. Boxed areas are shown in detail in the inset.

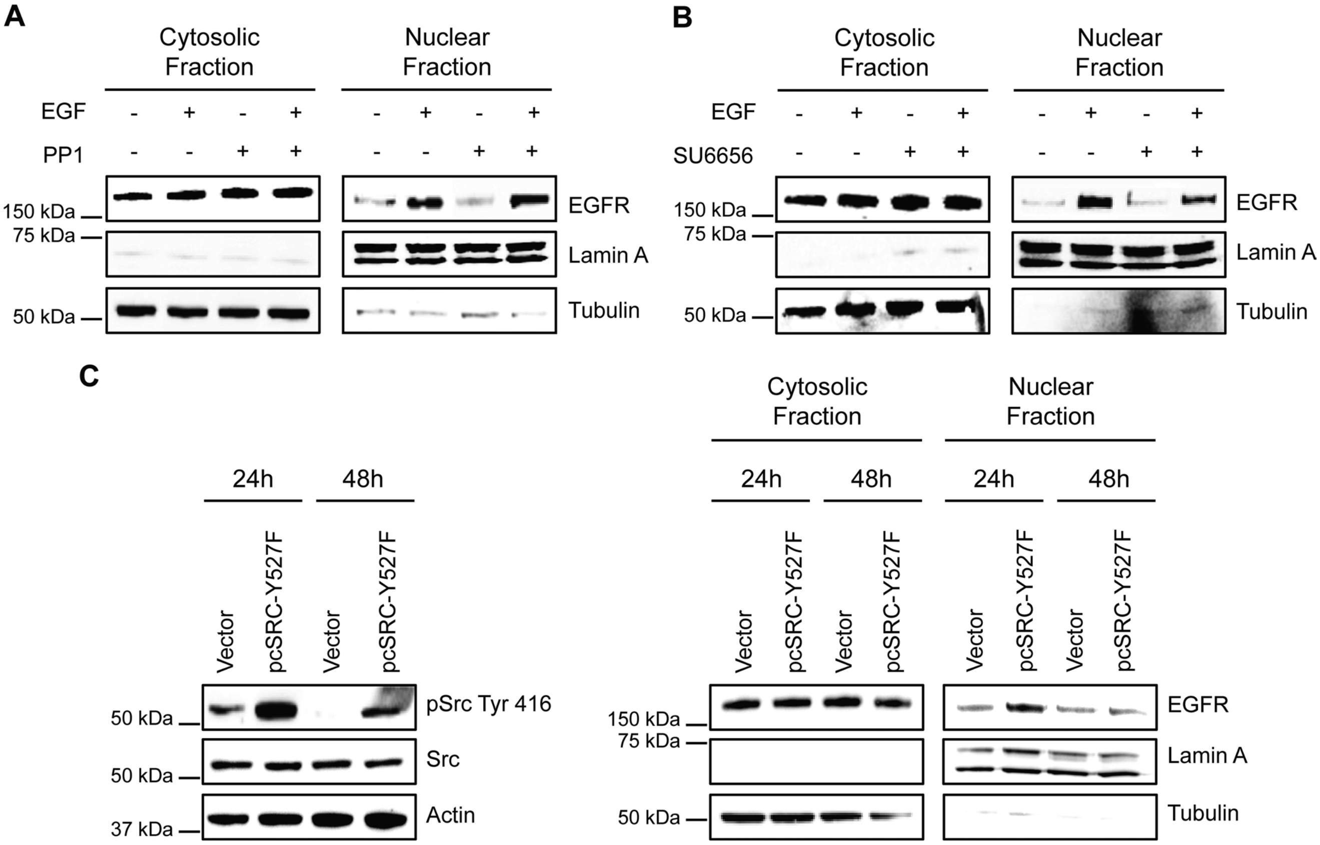

Supplementary Figure 9: SRC family kinases play a pivotal role in EGFR nuclear translocation. (**A**, **B**) Immunoblotting analysis of EGFR localization in A549 exposed for 10 min to 25ng/ml EGF with or without 10 μM PP1 or SU6656. Tubulin and Lamin A were used as loading control for cytosolic and nuclear fraction, respectively. (**C**) A549 cells were transfected with Vector control or a plasmid bearing constitutive-active SRC (pcSRC-Y527F) for 24 h and 48 h. Immunoblotting analysis of SRC phosphorylated on Tyr 416 in A549 transfected for 24 h or 48 h was performed as control for plasmid expression and SRC activation. Actin was used as loading control (left panel). EGFR expression at 24 h or 48 h in cytosolic and nuclear fraction in A549 transfected with pcSRC-Y527F (right panel).

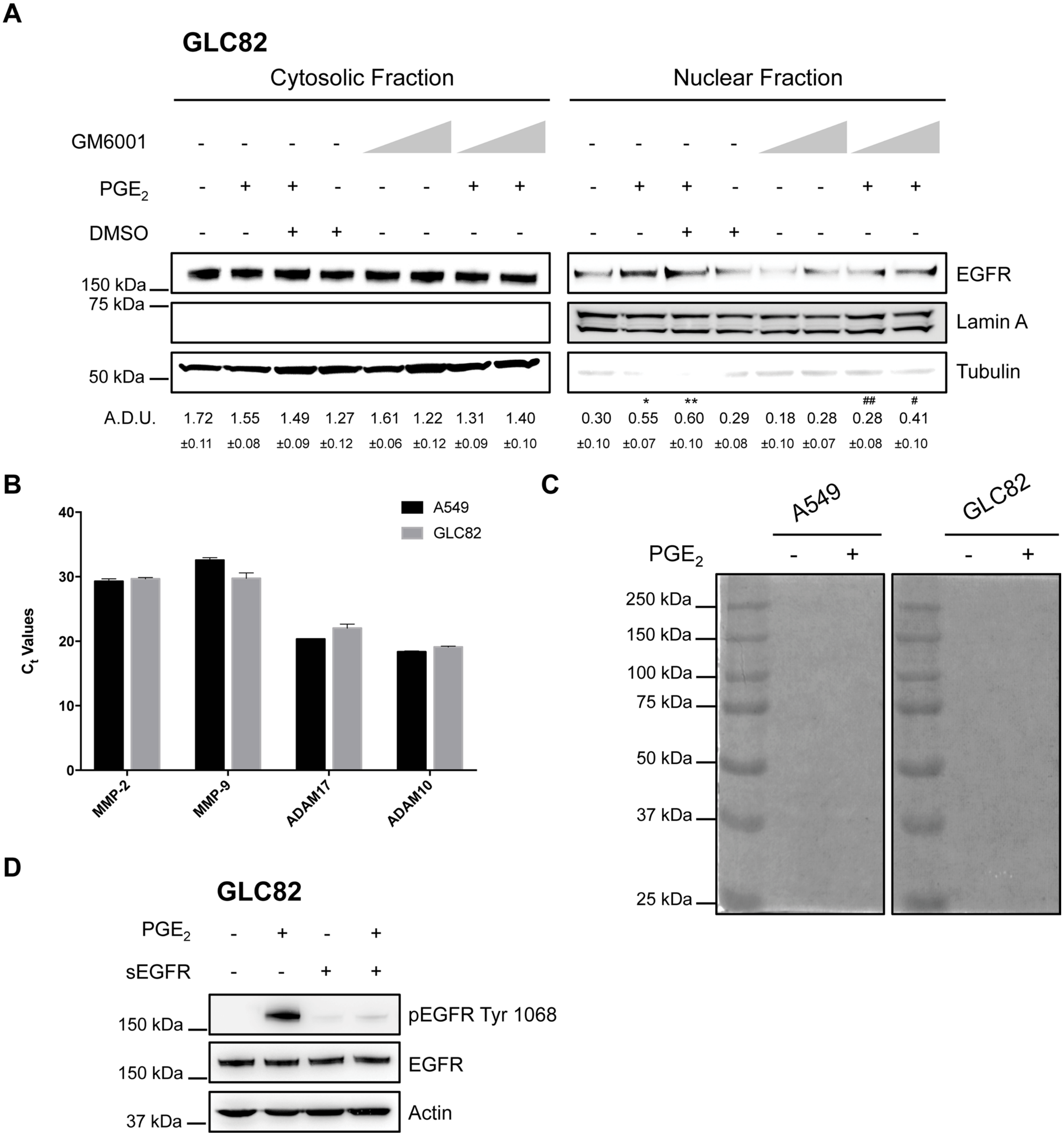

Supplementary Figure 10: PGE_2 acts via ADAMs-mediated shedding of EGF-like ligands to promote EGFR activation and nuclear translocation in GLC82. GLC82 were starved overnight and then pre-treated with 10 µM or 25 µM GM6001 before challenge with 1µM PGE_2 for 60 min. DMSO, matching the solvent concentration of 25µM GM6001, was used as a control. (**A**) Immunoblotting analysis of EGFR expression in cytosolic and nuclear fraction of GLC82 treated as described above. Immunoblotting quantification was expressed in A.D.U. (arbitrary density unit) and as mean ± SEM. $^*p < 005$, $^{**}p < 0.01$ vs Ctrl; $^{\#}p < 0.05$, $^{\#\#}p < 0.01$ vs PGE_2+DMSO. (**B**) qRT-PCR analysis of basal mRNA expression for MMP-2, MMP-9, ADAM17 and ADAM10 in A549 and GLC82. Results are presented as mean of Ct values ± SEM of two independent experiments. (**C**) A549 and GLC82 cells were starved overnight and then exposed to 1 µM PGE_2 for 30 min. MMP-2 and MMP-9 activation was evaluated by gelatin zymography. The gels shown are representative of two obtained with similar results. (**D**) Immunoblotting analysis of EGFR phosphorylation on Tyr1068 and total EGFR expression in GLC82 exposed to 1 µM PGE2 and 50 µg/ml sEGFR was performed.

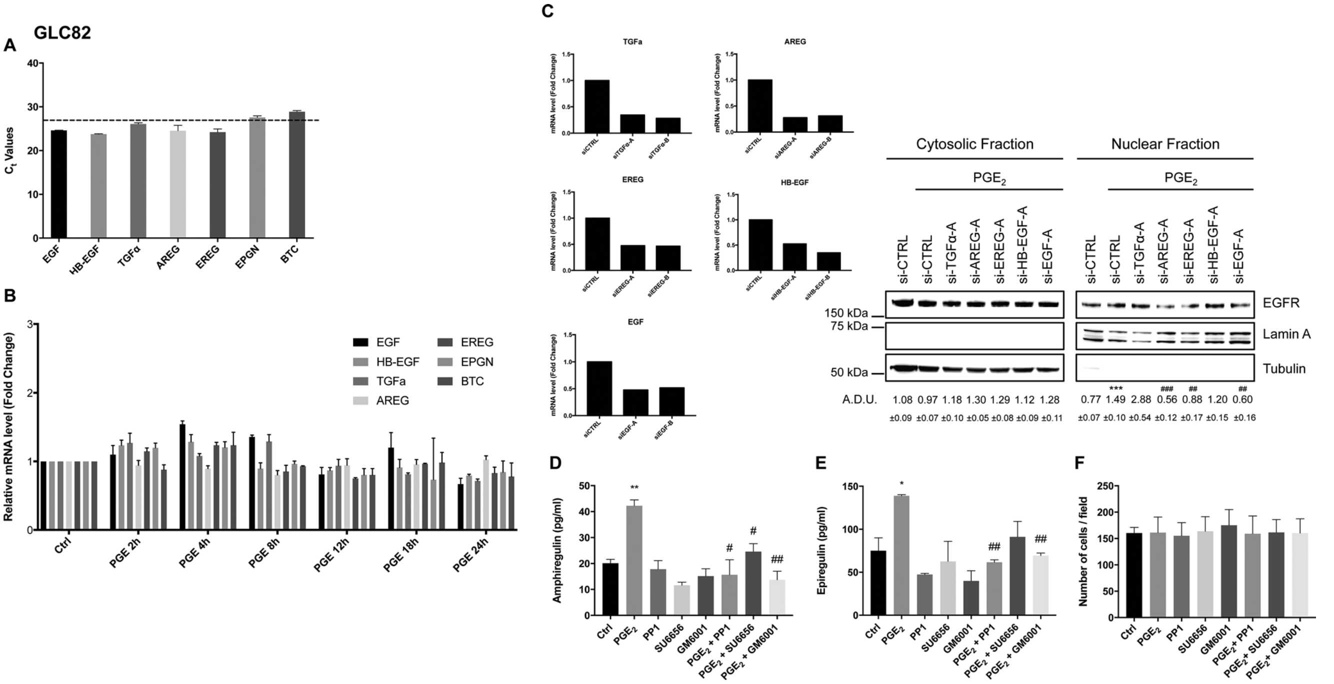

Supplementary Figure 11: EGFR ligands mediates PGE_2-dependent EGFR nuclear translocation in GLC82. (**A**) qRT-PCR analysis of basal mRNA expression for EGFR ligands in GLC82. Results are presented as mean of Ct values ± SEM of two independent experiments. (**B**) GLC82 cells were starved overnight and then treated with 1 µM PGE_2 for 2, 4, 8, 12, 18 and 24 h. RNA was isolated and analyzed by qRT-PCR for EGFR ligands. The data are presented as mean of fold change ± SEM of three independent experiments, relative to non-treated cells (Control), which were assigned to 1. (**C**) mRNA expression analysis of EGFR ligands by qRT-PCR in GLC82 silenced for 48 h for AREG, EREG, TGF-alpha, HB-EGF and EGF with two different oligos (siRNA A and B) (left panel). EGFR expression analysis by immunoblotting of cytosolic and nuclear fraction in GLC82 silenced for EGFR ligands (right panel). Tubulin and Lamin A were used as loading control for cytosolic and nuclear fraction respectively. Immunoblotting quantification was expressed in A.D.U. (arbitrary density unit) and as mean ± SEM. ****p* < 0.001 vs siCTRL; ## *p* < 0.01, $^{###}p$ < 0.001 vs siCTRL+PGE_2. Similar data were obtained with siRNA-B (Data not shown). (**D**, **E**) ELISA for AREG (D) and EREG (E) in conditioned media from GLC82 exposed for 60 min to PGE_2 (1 µM), PP1 or SU6656 (10µM) or GM6001 (10 µM), or PGE_2 + PP1, PGE_2 + SU6656 and PGE_2 + GM6001. Data are reported as pg/ml. **p* < 0.05, ***p* < 0.01 vs Ctrl (control condition); $^{#}p < 0.05$, $^{##}p < 0.01$ vs PGE2. (**F**) Cell number in wells exposed to conditions described above for 60 min to obtain the conditioned media.

Supplementary Table 1: List of siRNA sequences

Target	Target Sequence (5′-3′)
EP3#1	AAGAAAGA TCCTTCTTCGAAA
EP3#2	CACGACCGAGACGGCCATTCA
TGFα-A	GACCCUAGCUUUAAGAGAA
TGFα-B	AAGCUAUACUGAUUAGAAA
AREG-A	GACAAUACGUCAGGAAAUA
AREG-B	UGACAGUAGUUUAUCAAAA
EREG-A	GCUUUGACCGUGAUUCUUA
EREG-B	CAGUCGUCGGUUCCACAUA
HB-EGF-A	CCAUGUCUUCGGAAAUACA
HB-EGF-B	GGACCCAUGUCUUCGGAAA
EGF-A	CTGGACTGATACAGGGATTAA
EGF-B	AACGATTGACTTCTTAACTGA

Supplementary Table 2: List of qRT-PCR primers

Name	Forward primer (5′-3′)	Reverse primer (5′-3′)
EGF	CATCCATTGGCAAAACCAG	AACACCAAGCAGTTCCAAGC
HB-EGF	TGGGGCTTCTCATGTTTAGG	CATGCCCAACTTCACTTTCTC
TGFα	CCTGGCTGTCCTTATCATCAC	GGCACCACTCACAGTGTTTTC
AREG	TTGACAGTAGTTTATCAAAAATTGCAT	CCTGACGTATTGTCTTCTAAGCTG
EREG	AAATGTAGAAACTTAAACACACCTTCC	TGTCAGGAAAATGAGCCCTAGT
EPGN	TTCAACGCAATGACAGCACT	GGTCCTTCTATGTTGTCAGCTTG
BTC	GATGGGAATTCCACCAGAAG	TCCGCTTTGATTGTGTGGT
RPL19	GATGCCGGAAAAACACCTTG	TGGCTGTACCCTTCCGCTT
AURKA	GCAGATTTTGGGTGGTCAGT	TAGTCCAGGGTGCCACAGA
MYBL2	AAGGTCAAATGGACCCATGA	AGTCCTGCTGTCCAAACTGC
NOS2	GCTGCCAAGCTGAAATTGA	GATAGCGCTTCTGGCTCTTG
PTGS2	GCTTTATGCTGAAGCCCTATGA	TCCAACTCTGCAGACATTTCC
MYC	CACCAGCAGCGACTCTGA	CTGTGAGGAGGTTTGCTGTG
CCND1	GACCTTCGTTGCCCTCTGT	GGTTCAGGCCTTGCACTG
BCRP	TTCCACGATATGGATTTACGG	GTTTCCTGTTGCATTGAGTCC
TYMS	CCCAGTTTATGGCTTCCAGT	GCAGTTGGTCAACTCCCTGT
ADAM10	ACATAACTTTGGATCCCCACA	CCATTTTCTTTTTGACCCAAAT
ADAM17	GTGGATGGTAAAAACGAAAGCG	GGCTAGAACCCTAGAGTCAGG
MMP-2	CCCACTGCGGTTTTCTCGAAT	CAAAGGGGTATCCATCGCCAT
MMP-9	TTGACAGCGACAAGAAGTGG	GCCATTCACGTCGTCCTTAT
